# Phytochemical-Functionalized Smart Polymer Films: Antioxidant and Antimicrobial Strategies for Next-Generation Active Packaging

**DOI:** 10.3390/polym18212764

**Published:** 2026-11-06

**Authors:** Arulkumar Nagappan, Mahenthiran Ramasamy, Radha Arulkumar, Arvind Kumar Shukla, Sethuraman Veeran

**Affiliations:** 1 Department of Pathology, Saveetha Medical College and Hospital, Saveetha Institute of Medical and Technical Sciences (SIMATS), Saveetha University, Thandalam, Chennai 602105, India; arulbiotechtnau@gmail.com; 2 Department of Microbiology, Dr. N.G.P Arts and Science College, (Affiliated to Bharathiar University), Coimbatore 641048, India; mahenthiran@drngpasc.ac.in; 3 PG & Research Department of Biotechnology & Bioinformatics, Holy Cross College (Autonomous), (Affiliated to Bharathidasan University), Tiruchirappalli 620002, India; radharul15@gmail.com; 4 School of Biomedical Convergence Engineering, Pusan National University, Yangsan 50612, Republic of Korea; 5 Department of Orthopaedics, Saveetha Medical College and Hospital, Saveetha Institute of Medical and Technical Sciences (SIMATS), Chennai 602105, India

**Keywords:** active packaging, phytochemicals, biopolymers, smart packaging, antioxidant films, polyphenols, essential oils, food preservation, sustainable materials

## Abstract

The worldwide shift toward sustainable food systems demands packaging solutions that are compatible with the environment, food safety and shelf-life extension. Phytochemical-functionalized smart polymer films have emerged as promising multifunctional active packaging systems with antioxidant and antimicrobial properties. This review critically examines the molecular mechanisms, fabrication strategies, structure-release-performance relationships, applications, and translational challenges of phytochemical-based smart polymer films. Recent advances in incorporating polyphenols, essential oils, curcuminoids, tocopherols, and related bioactives into polysaccharide-, protein-, and bio-based polyester matrices through direct blending, encapsulation, cyclodextrin inclusion, electrospinning, and hybrid nanoparticle systems are evaluated. Phytochemicals can scavenge reactive oxygen species, chelate pro-oxidant metals, and interrupt radical chain reactions, while smart systems enable controlled or stimuli-responsive release triggered by pH, humidity, temperature, or spoilage volatiles. These designs influence antioxidant and antimicrobial activity, oxygen and water-vapor barrier properties, UV protection, and mechanical performance. Applications across meat, fish, dairy, fruits, vegetables, and high-fat foods demonstrate potential for delaying oxidation, suppressing microbial growth, and maintaining sensory and nutritional quality. This review synthesizes phytochemical–matrix–processing–release–performance relationships into a qualitative, application-oriented design framework, emphasizing measurable parameters, formulation-selection criteria, and validation in the intended food and storage environment.

## 1. Introduction

Population growth, urbanization, climate change and increased consumer demand for safe, nutritious and minimally processed foods are posing unprecedented challenges to the global food system. A large proportion of food produced for human consumption is lost or wasted along the supply chain. Major losses occur due to post-harvest deterioration by microbial spoilage and oxidative degradation, particularly in perishable commodities such as meat, fish, dairy, fruits and vegetables [1,2]. The Food and Agriculture Organization (FAO) and the United Nations Environment Program (UNEP) estimated recently that about 13% of food is lost between harvest and retail, and an additional 19% of food, or 1.05 billion tons, is wasted at the retail, food service and household levels [3,4]. These figures point out that nearly one third of all food produced world-wide is never consumed by consumers, a huge ethical and economic concern but also a significant environmental burden in terms of resource depletion, greenhouse gasses and waste disposal [5,6,7].

Conventional plastic packaging, produced from petroleum-based materials, has successfully provided mechanical protection and barrier properties, but is increasingly challenged by its environmental footprint, persistence in the ecosystem and limited functionality beyond passive containment [8,9]. The packaging industry, in turn, is moving toward sustainable, bio-based and multifunctional materials that will not only reduce environmental impact but will actively contribute to food preservation and maintenance of quality. In this context, active packaging, a packaging system that interacts with food or the headspace to prolong shelf life, improve safety or improve sensory and nutritional properties, has become a game changer [10]. Active packaging strategies such as antioxidant and antimicrobial films have attracted much attention because of their ability to retard lipid oxidation, inhibit microbial growth and preserve color, flavor and texture during storage [11,12].

In the early days of active packaging, synthetic additives or inorganic antimicrobials incorporated in conventional polymers were commonly used. However, the increasing consumer demand for ‘clean-label’ products and the regulatory and safety issues associated with synthetic compounds have spurred interest in natural, plant-derived bioactives as functional ingredients in packaging materials [13]. Polyphenols, flavonoids, tannins, essential oils, curcuminoids and tocopherols are phytochemicals with a wide range of antioxidant, antimicrobial, anti-inflammatory and UV-absorbing properties, making them attractive candidates for next generation active packaging [14,15]. These compounds are obtained from herbs, spices, fruits, vegetables, and agro-industrial waste, and are in accordance with the principles of circular economy, as they confer high-value functionalities to waste streams [16,17].

At the same time, progress in polymer science has allowed the creation of smart and stimuli-responsive materials that can adjust the release of the active compounds in response to environmental factors, including pH, humidity, temperature, or the presence of spoilage metabolites [18,19,20]. These intelligent polymer systems, in combination with phytochemicals, provide opportunities for on demand, synchronized delivery of antioxidants and antimicrobials at exactly the right time to respond to the beginning of oxidation or microbial growth in specific food matrices. The application of nanotechnology, such as the use of nanoparticles, nanofibers, nanocomposites, and inclusion complexes, has further improved the dispersion, stability, and controlled release of phytochemicals in polymer matrices and has often increased the mechanical strength, barrier performance, and other functionalities such as UV shielding [21,22,23]. The literature remains fragmented across phytochemistry, polymer/materials science, and food preservation. Existing reviews mainly address plant-extract bioactivity, biopolymer-film fabrication, or food-packaging applications separately, with limited integration of phytochemical structure, polymer architecture, release kinetics, and real-food performance. Although advances in nanocomposites, stimuli-responsive systems, and antioxidant films have emerged, an integrated, application-oriented framework for the rational design of phytochemical-functionalized smart packaging remains lacking.

Active packaging, freshness indicators and stimuli-responsive release systems are different but potentially complementary functions. These can be used for quality maintenance (e.g., antimicrobial or antioxidant); scavenging of undesirable substances in or on the food or in the surrounding atmosphere (e.g., ethylene scavenging); and other protective packaging purposes (e.g., oxygen scavenging). Freshness indicators give a detectable signal of the changes in food quality (e.g., color changes due to pH change or volatiles associated with spoilage). Stimulus-responsive delivery systems, in contrast, control the release of an active compound upon exposure to a given stimulus (e.g., pH, temperature, humidity, or enzyme). Therefore, pH-induced color change is an appropriate optical response but does not constitute evidence for triggered antimicrobial delivery. Thus, release must be shown as a stimulus-dependent change in the amount or rate released compared with appropriate comparators. Therefore, it is inadvisable to describe a colorimetric antimicrobial film as an active-intelligent multifunctional system in the absence of a similar demonstration of stimulus-dependent release.

Other recent reviews have addressed natural extracts in active polymer food packaging systems [24] and advances in release-type systems in terms of bioactive selection and incorporation technologies, polymer matrix types, and food-preservation applications [25]. This review builds on the contributions by situating the evidence in a structure–release–performance framework: bioactive stability and transport as a function of phytochemical chemistry and polymer architecture and the consequences of the former on functional performance in food systems. This is a deliberate juxtaposition of design trade-offs and not a deliberate omission of reviews on the individual materials or mechanisms involved. These relations are then expressed as qualitative design principles for phytochemical–material compatibility, stability during processing, matching of release and degradation, matching of barriers and mechanics, specific food validation, and balancing sensory properties, migration, and mechanism. Decisions are based on these principles, but employment of a packaging system requires validation by bio-based material–food–storage combination.

## 2. Literature Search Strategy

This narrative review was informed by literature searches in Web of Science, Scopus, PubMed, and Google Scholar. The initial searches were performed on 30 September 2026 and the search in PubMed was updated on 23 October 2026. The articles were searched from 1 January 2022 to 23 October 2026. Search terms were combined using the Boolean operators and were “phytochemical,” “polyphenol,” “essential oil,” “natural antioxidant,” “active packaging,” “smart packaging,” “intelligent packaging,” “biopolymer film,” “edible film,” “biodegradable packaging,” “controlled release,” “stimuli-responsive packaging,” “food preservation,” and “food packaging.” Additional publications were screened from reference lists of relevant research articles, reviews, and book chapters. Relevant publications included phytochemical-functionalized polymer films, coatings, or other carriers related to active or intelligent food packaging. Original research articles were included if they addressed any of the phytochemical–polymer interactions, incorporation and stabilization, release, film properties, or food-preserving performance.

Relevant review articles were included to contextualize developments in the field, and publications on migration, safety, regulatory requirements, scalability, or environmental performance were included to underpin the translational discussion. Studies that focused exclusively on the extraction or biological activity of phytochemicals without discussing their safety- or preservation-related design within packaging materials or food preservation were omitted from the principal synthesis. Drug delivery studies were included if the mechanisms were related to the stimulus-response systems in question (evidence of pure delivery derived from food-packaging applications is different). After removing duplicate records, publications were evaluated based on the topical scope of the review and their criteria of inclusion. Literature was selected and synthesized qualitatively based on relevance to the narrative scope of the review. The search and selection process was intended to support a critical narrative synthesis rather than an exhaustive systematic review. Complete counts of records identified, screened, and excluded were not retained; consequently, the completeness of coverage and the distribution of studies across food categories cannot be quantified. The organization by food category should therefore be interpreted as a thematic presentation of selected evidence, not as a ranking of research activity or formulation effectiveness.

## 3. Phytochemicals as Antioxidant and Antimicrobial Agents

Phytochemicals are bioactive secondary metabolites derived from plants. They have been extensively studied as natural multifunctional additives for active packaging owing to their strong antioxidant, antimicrobial, anti-inflammatory and UV-absorbing properties. These compounds include polyphenols, flavonoids, tannins, essential oils, curcuminoids and tocopherols. They provide a sustainable alternative to synthetic preservatives and meet consumer demand for clean-label and environmentally friendly food systems. In the use of polymers in packaging, phytochemicals are not just inert ingredients but active functional agents to reduce the rate of oxidative deterioration, impede microbial growth and maintain sensory and nutritional quality in different food matrices [24,25].

### 3.1. Classes of Phytochemicals Used in Active Packaging

Polyphenols are the largest and most studied class of phytochemical for active packaging applications. This group includes simple phenolic acids (e.g., gallic acid, caffeic acid, and ferulic acid), flavonoids (e.g., quercetin, catechin, and anthocyanins), stilbenes (e.g., resveratrol) and lignans all having one or more aromatic rings with hydroxyl groups. Flavonoids are characterized by the C6-C3-C6 skeleton and are divided into flavonols, flavones, flavanones, isoflavones and anthocyanins, with different antioxidant potentials and reactivity patterns [26,27,28]. Tannins, such as condensed proanthocyanidins and hydrolysable ellagitannins, are high-molecular-weight polyphenols that can form strong complexes with proteins and polysaccharides, affecting film structure and release behaviour [29,30].

Another major group of phytochemicals used in active packaging is essential oils (EOs). These lipophilic volatile extracts are abundant in terpenoids (e.g., limonene and α-pinene), phenolics (e.g., carvacrol, thymol, and eugenol), aldehydes (e.g., cinnamaldehyde), and alcohols (e.g., linalool and geraniol). Thus, EOs, such as oregano, thyme, clove, rosemary, and cinnamon, which have exhibited a wide range of antimicrobial and antioxidant activities, are highly suitable for use in biopolymer matrices [31,32,33]. Curcuminoids are lipophilic polyphenolic pigments isolated from the rhizomes of turmeric (*Curcuma longa*). The major constituents are curcumin, demethoxycurcumin and bisdemethoxycurcumin. The number of methoxy substituents on the aromatic rings of these compounds varies. The main curcuminoid is curcumin, which due to its antioxidant properties and pH dependent color change is of particular interest in active and intelligent packaging. It can therefore be incorporated in polymer matrices to enable food preservation as well as visual freshness sensing. However, its poor aqueous solubility makes formulation and encapsulation relevant points to consider during film development. The lipid soluble members of the vitamin E family are called tocopherols and include α-, β-, γ- and δ-tocopherol, which differ in the number and position of methyl groups on the chromanol ring. They consist of a phenolic chromanol head group and a saturated hydrophobic side chain. The phenolic hydroxyl group can donate a hydrogen atom to lipid peroxyl radicals and interrupt the chain of lipid oxidation. Thus, tocopherols are important antioxidant additives for packaging materials for the protection of lipid-containing foods. Their effectiveness depends not only on vitamin E activity, but also on the form, concentration and surrounding food or material environment of the tocopherol [34,35,36,37].

### 3.2. Molecular Mechanisms of Antioxidant Action

The antioxidant function of phytochemicals in packaging systems is mainly due to their ability to scavenge reactive oxygen species (ROS) and interrupt radical chain reactions responsible for lipid oxidation, protein carbonylation and pigment degradation in foods [38,39]. The antioxidant activity of polyphenols and flavonoids is based on three main mechanisms: hydrogen atom transfer (HAT), single-electron transfer (SET) and transition metal chelation (TMC) [40,41]. In the HAT mechanism, a phenolic hydroxyl group donates a hydrogen atom to a free radical (*R*•), yielding a stable, non-radical species (RH) and a relatively stable phenoxyl radical (ArO•) that is unable to propagate oxidative damage. This process can be described as follows:

*ArOH* + *R*• → *ArO*• + *RH*

The HAT efficiency is dependent on the bond dissociation enthalpy (BDE) of the O-H bond, with lower BDEs corresponding to higher reactivity and higher antioxidant potency. The radical-scavenging ability of flavonoids increases with the number of hydroxyl groups and especially the ortho-dihydroxy (catechol) structure on the B-ring, which stabilizes the phenoxyl radical through resonance [41,42]. Polyphenols can donate an electron to oxidizing species such as superoxide anion (O2-^−^) or peroxyl radicals (ROO-), which is often followed by proton transfer, in the SET mechanism, thus reducing the oxidant and generating a radical cation that is then stabilized [40]. In polar environments and at physiological pH, the ionization of phenolic groups facilitates electron donation and this pathway is particularly important.

A third, complementary, antioxidant strategy is the chelation of transition metals. Polyphenol anions, formed by deprotonation of hydroxyl groups, can chelate pro-oxidant metal ions such as Fe2+ and Cu2+ and thus prevent these metal ions from catalyzing the Fenton and Haber–Weiss reactions that produce highly reactive hydroxyl radicals (-OH) [43,44]. Such metal-chelating capacity is of particular importance in meat, fish and dairy products where heme iron and copper ions are involved in the promotion of lipid peroxidation. Essential oil constituents, particularly phenolic monoterpenes such as carvacrol, thymol and eugenol, serve as strong radical scavengers as well by donating hydrogen atoms from their phenolic –OH groups to lipid peroxyl radicals (ROO-) thereby terminating propagation chains [45]. Moreover, some EO constituents are capable of regenerating other antioxidants (e.g., tocopherols) or synergistically act with polyphenols to enhance the total antioxidant capacity [46,47].

The antioxidant mechanism of phytochemicals is dependent on their molecular frameworks and chemical environment in which they are present. Common radical-scavenging pathways are identified in several classes, though the relative contributions of hydrogen-atom transfer, electron transfer, and metal chelation vary between individual compounds. In the discussion below, the main mechanisms associated with the phytochemical groups considered in this review are distinguished.

#### 3.2.1. Polyphenols: Phenolic Acids, Flavonoids, and Tannins

Polyphenolic antioxidants act through overlapping pathways, the relative importance of which is dependent on their hydroxyl substitution, conjugation, ionization state and surroundings. Phenolic acids and flavonoids are capable of quenching radicals through hydrogen-atom transfer or electron-transfer pathways to produce relatively stabilized phenoxyl species. Transition metals can also influence the oxidation of compounds with appropriate oxygen-containing coordination sites, catalyzed and bound to the metal. It should not be assumed that these mechanisms are equally contributory for each polyphenol. A subgroup of this group is flavonoids, e.g., quercetin, which have radical-scavenging activity and metal-binding capacity. Hydroxyl and carbonyl group positioning influences the activity. Tannins contain several phenolic sites able to scavenge radicals and bind metal ions, but their interactions with proteins and other matrix components may affect the accessibility of these sites. Therefore, antioxidant activity should be interpreted at the level of the compound and formulation, and not simply by the fact that it belongs to the polyphenol class.

#### 3.2.2. Essential-Oil Constituents

Many of the constituents of essential oils are antimicrobial because they partition into microbial membranes. Phenolic constituents such as thymol, carvacrol and eugenol have demonstrated disruption of membrane organization, increased permeability and impairment of ion gradients and membrane-associated functions. The contribution of each pathway varies depending on the constituent and microorganism. Aldehyde-containing constituents such as cinnamaldehyde should be separated from phenolic constituents as their functional groups will enable different interactions with microbial targets. Hence, the activity of an essential oil is not of any universal mechanism but a function of its composition and interaction of its constituents.

#### 3.2.3. Curcuminoids

Curcumin has demonstrated antimicrobial activity through several established mechanisms, including the disruption of the bacterial membrane, interference with bacterial division, and biofilm-associated processes and virulence. The effects depend on the organism, the concentration and the formulation. In photodynamic systems, a different mechanism is involved: curcumin acts as a photosensitizer and forms reactive oxygen species that damage microbial components under appropriate irradiation. Thus, light-dependent antimicrobial activity should be separated from the activity measured in the absence of light and the results obtained for curcumin should not be directly extrapolated to all curcuminoids.

#### 3.2.4. Tocopherols

Tocopherols are mainly considered in terms of their antioxidant contribution and should not be necessarily assumed to have the membrane-disrupting or enzyme-inhibiting mechanisms described for other phytochemicals. In multicomponent films, the antimicrobial activity should be attributed with caution, since the polymer matrix, co-incorporated bioactives or nanoparticles can also contribute. The antimicrobial mechanism specific to tocopherol should be proposed only if supported by appropriate experimental evidence.

### 3.3. Mechanisms of Antimicrobial Action

Phytochemicals, in addition to having antioxidant properties, have a wide spectrum of antimicrobial activity against spoilage and pathogenic microorganisms such as Gram-positive and Gram-negative bacteria, yeasts and molds. Polyphenols and essential oils have multiple mechanisms of action and often act synergistically to affect the viability of microorganisms. Phenolic compounds and essential oils are amphiphilic or hydrophobic, and therefore they mainly act on the cytoplasmic membrane. These compounds interact with the lipid bilayer and embed into the membrane, causing structural destabilization, increased fluidity and increased permeability [48]. This perturbation of the membrane causes leakage of intracellular components (ions, ATP, and nucleic acids), collapse of the membrane potential, and, ultimately, cell lysis [49]. EOs rich in phenolics such as carvacrol and eugenol and EOs that contain thymol are particularly effective in destabilizing bacterial membranes as they are capable of establishing hydrogen bonds with the phospholipid head groups and disrupting lipid packing [48].

In addition to damage to cell membranes, phytochemicals can influence critical cellular processes. Polyphenols and flavonoids have been reported to inhibit key enzymes involved in energy metabolism (e.g., ATPases), nucleic acid synthesis (e.g., DNA gyrase) and cell wall biosynthesis (e.g., transpeptidases). Specific compounds, such as catechins and proanthocyanidins, can bind to microbial surface proteins and adhesins, thereby inhibiting attachment and biofilm formation on food surfaces [50]. Essential oils and their constituents also exhibit anti-quorum sensing activity, disrupting cell–cell communication systems governing virulence factor production and biofilm maturation in pathogens such as Pseudomonas aeruginosa and Staphylococcus aureus [48]. Because they contain ergosterol in their membranes instead of cholesterol, fungal cells are particularly susceptible to phenolic EOs that interact with ergosterol and compromise membrane integrity [49]. The selective toxicity is the basis of the effectiveness of the phytochemical-based packaging toward molds such as Aspergillus, Penicillium and Botrytis, which are the main causes of post-harvest spoilage of fruits, vegetables and bakery products.

The antimicrobial effects of phytochemicals vary with their molecular structures, concentrations, target microorganisms, and formulation conditions. Although some compounds share mechanisms such as membrane disruption, their specific targets and relative contributions differ. The following discussion distinguishes the reported mechanisms associated with the principal phytochemical groups considered in this review.

### 3.4. Structure–Activity Relationships

The antioxidant and antimicrobial potency of phytochemicals is closely related to their molecular framework, which determines their reactivity, solubility, and interaction with polymer matrices and food components. Number and position of the hydroxyl groups, degree of conjugation, and the presence of electron-donating or -withdrawing substituents are the main structural factors that determine the antioxidant activity of polyphenols and flavonoids [38]. Flavonoids with a catechol moiety (3′,4′-dihydroxy) on the B-ring, such as quercetin and luteolin, have higher radical scavenging activity compared to monohydroxylated analogs because of increased resonance stabilization of the phenoxyl radical [38]. Glycosylation of flavonoids improves their water solubility but often decreases their antioxidant activity by sterically hindering the accessibility of hydroxyl groups [51].

Essential oil phenolic constituents (carvacrol, thymol, and eugenol) generally show better antimicrobial and antioxidant activities than non-phenolic terpenes (limonene and α-pinene) due to their hydrogen atom-donating capacity and membrane disruption capacity [52,53,54]. The reactivity is further enhanced by the presence of an allyl or propenyl side chain (e.g., in eugenol and isoeugenol) due to the resonance stabilization of radical intermediates [38]. Aldehydes like cinnamaldehyde are known to possess strong antimicrobial activity due to formation of a Schiff base with microbial proteins, but their antioxidant activity is comparatively lower than phenolic EOs [55,56]. The effectiveness of phytochemicals in packaging systems is also influenced by lipid solubility. Lipophilic antioxidants like tocopherols and curcumin are effective in protecting high-fat foods such as meat, fish and oils from lipid peroxidation, while hydrophilic polyphenols (e.g., gallic acid and catechins) are more suitable for aqueous or high-moisture foods such as fruits, vegetables and dairy [38]. This dichotomy emphasizes the necessity of matching phytochemical polarity with the target food matrix for maximum protection.

Therefore, the mechanisms of the phytochemicals should not be preassigned but considered in the food environment in the choice of antioxidants. Phenol radical trapping may occur via HAT in non-ionizing lipids, but the phenol deprotonation in aqueous or ionizing media in the food system may favor electron-transfer pathways after phenol deprotonation, and the individual pathways’ relative contribution depends on pH, penetrability of solvent molecules, and reactive species and not only on polarity [57]. If so, then the antioxidant must be present at the oxidation site (e.g., lipid–water interfaces), and activity in an aqueous assay cannot be uncritically extrapolated to oils, emulsions, or meat [58]. Cut-off values are not generalizable; temperature and water activity affect reactant mobility, metal availability, and oxidation kinetics [59]. Non-heme iron chelation will differ from heme association with protein (meat), and an aqueous iron-chelation assay will not account for or predict protein/s protection. For example, caffeic acid had strong inhibition of perch-hemoglobin-catalyzed lipid peroxidation in washed muscle held at low temperature, whereas a pH 5.8/25 °C model system would have been misleading as to its effect on hemoglobin oxidation [60]. Thus, the formulation of proteins for application should be based on release and partitioning measurements and protein-containing controls and primary and secondary oxidation end-points at the storage conditions of interest rather than screening assays to postulate a dominant mechanism [58].

### 3.5. Implications for Packaging Design

Knowledge of phytochemical chemical diversity and mechanism of action of bioactivity is a prerequisite for rational design of active packaging systems. In choosing a suitable phytochemical or a combination thereof, it is necessary to take into account not only its intrinsic antioxidant and antimicrobial properties, but also its compatibility with the polymer matrix, stability during processing and storage, release kinetics in the target food environment and possible sensory effects (color, odor, and taste) on the packaged product [61]. For instance, anthocyanins are potent antioxidants that are pH sensitive, and may change colors in different food environments that can be useful for intelligent packaging applications but can also limit their application to products where color stability is of utmost importance [62].

Furthermore, the synergistic effects between different classes of phytochemicals, such as polyphenol–tocopherol regeneration or EO–polyphenol membrane disruption, can be used for the design of multifunctional films with enhanced efficacy at lower additive concentrations, thus decreasing potential sensory changes and regulatory problems [61,63]. These structure–function insights form the basis for the subsequent discussion of polymer matrices and incorporation strategies where the physicochemical properties of phytochemicals need to be carefully matched with material selection and processing techniques for optimal performance.

## 4. Polymer Matrices for Active Film Fabrication

The choice of polymer matrix is a significant factor in determining structural, functional and environmental properties of active packaging films functionalized with phytochemicals. Good film-forming ability, mechanical integrity, barrier properties to oxygen and water vapor, compatibility with bioactive compounds and preferably biodegradability and renewability are characteristics of ideal matrices for sustainability goals. Biopolymers from polysaccharides, proteins and bio-based polyesters have become important candidates due to their abundance, edibility and tunable physicochemical properties [64,65,66]. This section reviews the main groups of biopolymer matrices used in active packaging, with an emphasis on their advantages and disadvantages and the possibilities of improving their properties.

### 4.1. Polysaccharide-Based Matrices

Polysaccharides used in food-packaging films and coatings are obtained from plant, algal, microbial, and animal-associated sources. Examples that are often used include starch, cellulose and derivatives thereof, pectin, alginate, carrageenan, chitosan and gums derived from microbes or plants. Their suitability for packaging is dependent on molecular weight, chain architecture, distribution of functional groups and the conditions used for film formation. Hydroxyl-rich chains allow intermolecular hydrogen bonding. Materials such as chitosan, alginate, and pectin have charged groups, which can further promote electrostatic interactions and ionic cross-linking. These properties allow for the development of continuous films and the incorporation of functional additives. However, their generally hydrophilic nature makes moisture sensitivity a major limitation, as water uptake can change mechanical strength, gas-barrier properties and the release of incorporated bioactives. Thus, their performance must be tested under the humidity and storage conditions relevant to the intended food application.

Polysaccharides are the most investigated class of biopolymers for active packaging applications due to their natural abundance, low cost, excellent film-forming ability and natural biodegradability [67,68]. The most common polysaccharides are chitosan, starch, cellulose and its derivatives, alginate, pectin, carrageenan and gums. All of them could be used to form continuous films through hydrogen bonding and chain entanglement [64,69]. Chitosan is a chitin-derived polysaccharide whose amino groups become protonated under acidic conditions. It exhibits a strong natural antimicrobial activity against bacteria, yeasts and molds. The antimicrobial action is attributed to the positive charge of chitosan, enabling it to interact with negatively charged microbial membranes [65,67,70]. Chitosan films have good oxygen barrier properties and mechanical strength, but they have high hydrophilicity and water-vapor permeability, which limits their application in high-moisture foods [64]. Chitosan combined with other polysaccharides such as starch, cellulose, and alginate or the incorporation of hydrophobic additives such as lipids, waxes, and nanoparticles has been demonstrated to enhance water resistance while keeping the antimicrobial activity [69,71].

Cheap and renewable polysaccharide starch is composed of amylose and amylopectin. Starch can form transparent and flexible films with moderate mechanical properties and good oxygen barrier properties [64]. However, native starch films are very hydrophilic and suffer from retrogradation, leading to brittleness and low water resistance. Common strategies to increase flexibility and moisture resistance involve chemical modification (e.g., acetylation and oxidation), physical blending with other biopolymers (chitosan and PVA), and plasticization with glycerol or sorbitol [68,69]. The reinforcement with nanocellulose has also been reported to enhance the tensile strength and water barrier properties of starch-based nanocomposites [64].

Cellulose and its derivatives such as microcrystalline cellulose (MCC), nanocellulose (CNC and CNF), carboxymethyl cellulose (CMC) and methylcellulose (MC) show extraordinary mechanical strength, thermal stability and oxygen barrier properties because of their strongly ordered crystalline structures and extensive hydrogen bond networks [64,67]. In particular, nanocellulose has received significant attention owing to its high aspect ratio and surface area as well as its ability to form percolating networks that enhance mechanical and barrier properties at low loadings [69]. However, cellulose-based films are hydrophilic by nature and thus require chemical modification (e.g., esterification and grafting) or combination with a hydrophobic polymer to improve water resistance [64].

Alginate, an anionic polysaccharide derived from brown seaweed, forms strong, flexible films with good oxygen barrier properties but poor water-vapor resistance [64]. To enhance the mechanical stability and reduce the solubility of alginate films, they are often crosslinked with divalent cations (Ca2+ and Ba2+) via ionic gelation. The electrostatic complexation of alginate with chitosan yielded films with enhanced mechanical strength, barrier properties and antimicrobial activity, which are suitable for fresh produce and seafood [65].

Pectin is a plant cell wall polysaccharide, usually extracted from fruit processing by-products, mainly citrus peel and apple pomace. Its film- and gel-forming behavior make it suitable for edible coatings and composite packaging films. The degree of esterification is a key formulation variable. Calcium-mediated cross-linking can be employed with appropriate pectin formulations to reinforce the network. Pectin systems are capable of transporting phytochemicals and regulating gas exchange at the food surface. However, their moisture sensitivity and limited mechanical robustness often leads to their combination with other materials through blending, cross-linking, inclusion of lipids or multilayer systems. Carrageenan and agar are seaweed polysaccharides used as gel-forming matrices for edible films and coatings. Carrageenan-based materials can form cohesive networks, but their properties are dependent on the type of carrageenan and formulation. Their affinity for water limits water-vapor resistance and makes formulation modification an important consideration for moisture-sensitive applications. Gums like gum Arabic, xanthan gum, and gellan gum offer further possibilities for coating formation, rheological control, and composite-film development. These materials are not interchangeable polysaccharides and must be chosen for their specific gel-forming, thickening and film-forming properties.

### 4.2. Protein-Based Matrices

The proteins used in food-packaging films and coatings are obtained from animal (gelatin, collagen, whey and casein) and plant (soy, maize zein, wheat gluten and pea protein) sources. Unlike polysaccharides, proteins contain various amino-acid side chains giving polar, charged and hydrophobic regions. Film formation occurs through the creation of intermolecular networks during processing and drying. Film formation is influenced by hydrogen bonding, hydrophobic interactions, electrostatic interactions, and, when present, disulfide bonding. The film continuity, flexibility, mechanical strength and interactions with phytochemicals are strongly dependent on the protein composition and processing conditions, respectively. Many protein films can be good barriers to oxygen and aroma compounds under the right conditions, but their effectiveness is often reduced by moisture uptake. Thus, their properties should be described as a function of the protein type, formulation and storage humidity rather than as general properties of all protein-based films.

Biopolymers derived from animal (gelatin, collagen, whey, and casein) and plant (soy, zein, pea, and wheat gluten) sources are protein-based and have unique advantages for active packaging, including excellent oxygen and aroma barrier properties, biodegradability, and the presence of functional groups (–NH2, –COOH, and –OH) that can promote interactions with phytochemicals and cross-linking agents [67,72,73]. Gelatin, derived from the partial hydrolysis of collagen, is one of the most common protein matrices owing to its excellent film-forming ability, transparency and biocompatibility [72,73]. Gelatin films have good mechanical properties and oxygen barriers, but are highly sensitive to moisture, and poor resistance to water vapor limits their practical applications [67]. These shortcomings may be overcome by approaches such as cross-linking with genipin, citric acid, or transglutaminase; blending with polysaccharides such as chitosan, agar, and starch; and integration of nanomaterials (Ag, ZnO, and TiO2) to improve barrier and antimicrobial properties [71,72]. More recently, gelatin has been obtained from non-mammalian sources (fish and poultry) to overcome religious and ethical issues related to bovine and porcine gelatin [73,74].

Zein is a prolamin protein from corn that forms hydrophobic, shiny films with good oxygen and oil barriers, which is very suitable for high-fat foods [64,65]. Zein films are brittle and need plasticization (glycerol and PEG) or blending with other biopolymers (chitosan and alginate) to improve flexibility and processability [67]. Moreover, zein’s hydrophobic nature enables it to encapsulate and retain lipophilic phytochemicals, such as essential oils and curcumin, thus improving the efficacy of antioxidants and antimicrobials [65].

Soy protein isolate (SPI) and whey protein films show good mechanical strength and oxygen barriers, but like other protein matrices, they are characterized by high hydrophilicity and moisture sensitivity [64]. Chemical modification (acylation and enzymatic crosslinking), thermal denaturation and blending with polysaccharides or lipids are common approaches to improve water resistance and mechanical performance [67]. Soy protein films supplemented with essential oils (clove and thyme) have shown enhanced antioxidant and antimicrobial activity, prolonging the shelf life of meat and bakery products [65].

Other matrix options are also available with casein, wheat gluten and pea protein. Casein is a milk-derived protein used in film and coating formulations, but its hydrophilicity can lead to high moisture uptake and reduced mechanical and barrier performance in humid conditions. Wheat gluten can form cohesive protein networks with useful barrier properties for oxygen, but moisture sensitivity is a disadvantage. Pea protein is an alternative plant-based protein, and its film performance depends on the protein extraction, processing conditions, plasticization and blending. The application of these proteins should not only be considered in terms of film functionality but also in terms of suitability for food contact, allergen management where appropriate and compatibility with the intended product and target consumer group. Accordingly, protein-matrix selection should balance film-forming ability, moisture response, processing requirements, and phytochemical compatibility rather than assuming that all protein sources provide equivalent packaging performance.

### 4.3. Biodegradable and Bio-Based Polyester Matrices

Biodegradable polyesters, including bio-based PLA and PHAs such as PHB and PHBV, as well as commonly petroleum-derived PBAT, are hydrophobic thermoplastic alternatives that have better mechanical strength, water resistance, and processability through conventional extrusion and thermoforming processes, compared to hydrophilic polysaccharides and proteins [64,67]. PLA is the most commercially developed biodegradable polyester synthesized by fermentation of renewable resources such as corn starch or sugarcane to lactic acid followed by polymerization [64]. PLA films have good clarity, good moisture barriers, and high tensile strength but are brittle, have poor oxygen barriers, and degrade slowly under ambient conditions [67]. Plasticizers (PEG and citrate esters), impact modifiers or other biodegradable polymers (PBAT and PHBV) added to PLA improve flexibility and toughness, while the inclusion of nanocomposites (clay, ZnO, and essential oils) improves the barrier and antimicrobial properties [67,68].

PHAs are microbial polyesters with properties comparable to traditional plastics (e.g., polypropylene); however, they are completely biodegradable in soil and marine environments [64,67]. Representative examples include polyhydroxybutyrate (PHB) and its copolymer poly(3-hydroxybutyrate-co-3-hydroxyvalerate) (PHBV). PHBV films, however, are expensive and brittle, and thus not suitable for broad application, despite their good mechanical strength, oxygen barriers, and UV resistance [67]. The addition of phytochemicals or nanoparticles in PHBV blended with PLA or PBAT was proven to improve flexibility, barrier performance, and active functionality [68]. PBAT is a biodegradable aliphatic–aromatic copolyester that is highly flexible, tough and processable, making it an attractive blending partner for brittle biopolymers such as PLA and PHBV [67]. Films based on PBAT integrated with essential oils or extracts of plants demonstrated prolonged antimicrobial and antioxidant properties, especially in fresh produce and bakery products [65,68].

### 4.4. Trade-Offs and Modification Strategies

Although biopolymer matrices have advantages, there are intrinsic trade-offs in mechanical strength, barrier performance, hydrophilicity, and processability. Polysaccharides and proteins are generally good oxygen barriers but weak water-vapor barriers, whereas bio-based polyesters have a superior moisture barrier but limited oxygen barrier and higher cost [64,67]. These constraints require strategic modifications to adapt biopolymer films to specific food applications, ensuring compatibility with phytochemical additives and optimizing overall packaging performance.

These conflicting properties are typically balanced by methods such as blending and composite formation. The combination of two or more biopolymers (such as chitosan with starch, gelatin with alginate, and PLA with PBAT) based on synergistic interactions enhances mechanical integrity, barrier properties, and functional performance [69,71]. For instance, chitosan–starch blends demonstrate enhanced water resistance and antimicrobial activity compared to individual polymers, and gelatin–alginate electrostatic complexes generate films with improved tensile strength and reduced water-vapor permeability [64,65]. Likewise, PLA–PBAT blends could overcome the brittleness of PLA while maintaining biodegradability and processability, suitable for thermoformed packaging applications [67].

Cross-linking is another effective way to increase the network density and decrease the hydrophilicity. Chemical crosslinkers such as genipin, citric acid, and dialdehyde polysaccharides, and physical methods such as heat and UV treatment, improve mechanical stability and water-vapor resistance by forming covalent or physical bonds between polymer chains [64,71]. Notably, Schiff base reactions between amino-rich biopolymers (e.g., chitosan and gelatin) and aldehyde donors have arisen as a dynamic covalent approach that not only improves barrier properties but also imparts stimuli-responsive behavior, enabling pH- or humidity-triggered release of active compounds [71].

The use of nanocomposites has attracted much interest because they can improve several properties simultaneously at low filler loadings. Nanofillers such as nanocellulose, clay, metal nanoparticles (e.g., Ag), and metal oxide nanoparticles (e.g., ZnO and TiO_2_) can enhance mechanical strength, barrier properties, and active functionality through reinforcement, interfacial interactions, and filler-specific mechanisms [68,69]. For instance, the inclusion of nanocellulose as reinforcement in starch or chitosan matrices has been demonstrated to decrease oxygen transmission by up to 45% and improve moisture resistance, due to the increased hydrophobicity and tortuosity [64].

Lipid incorporation is a targeted approach to overcome the high water-vapor permeability of hydrophilic biopolymers. The water resistance can be significantly improved by emulsification of lipids or waxes into polysaccharide or protein matrices or by developing bilayer films with a hydrophobic lipid layer while the oxygen barriers can be maintained [64,67]. Both approaches provide versatile ways to design biopolymer matrices that meet the multifaceted requirements of active packaging and sustainability standards either alone or in combination. The following section examines advanced integration methods for controlled release and delivery of phytochemicals within specifically designed matrices.

Protein and polyester behavior difference in release and phytochemical interaction also should be considered. Protein matrices contain charged, polar, and hydrophobic amino-acid residues capable of interacting with phytochemicals through H bonding, electrostatic and hydrophobic association, or covalent cross-linking that may promote retention at the expense of phytochemical release, and protein-network swelling and transport could be adjusted by hydration and pH change. For example, phenolic acid identity, matrix interactions, water activity, and simulant viscosity all affected release from gelatin/chitosan films [75]. Conversely, PLA and PHBV matrices are relatively hydrophobic and semicrystalline, making the prediction of phytochemical compatibility and/or release more complex, as the compound polarity, polymer crystallinity, glass-transition behavior, and processing history have a strong influence. Melt-processed PLA–PHBV films of phenolic acids showed slow release, with greater release in the less-polar food simulant, indicating that blending the polyester matrix does not guarantee proper release in (aqueous) food [76]. Optimization of release or exposure to thermal conditions in polyester systems may be achieved by encapsulation or application of coatings, multiple layers, etc., and these approaches have been described, e.g., employing antimicrobial PHBV layers coated with eugenol or oregano oil [77,78]. However, the entire formulation and intended use should be evaluated for regulatory compliance: proteins used in edible or food-contacting layers need to be evaluated for their ingredient status and allergens, and polyester monomers, additives, processing aids, migrants, and degradation products have to meet food-contacting authorization and migration criteria. Thus, protein and polyester matrices should not be seen as the same carriers or simply judged by their film-forming ability.

## 5. Incorporation Strategies and Nanostructuring

The effective incorporation of phytochemicals into polymer matrices is crucial for the functional efficacy, stability and release characteristics of active packaging films. The selection of the incorporation method depends on the physicochemical properties of the bioactive compound (polarity, volatility, and thermal stability), the polymer matrix (hydrophilicity, processing temperature, and solubility), and the specific application (type of food, storage conditions, and desired release profile) [79,80,81]. This section discusses major incorporation techniques such as direct blending, encapsulation systems, electrospinning and hybrid polyphenol-nanoparticle approaches, advantages and disadvantages and their effect on film properties.

### 5.1. Direct Blending and Solvent Casting

Direct blending is the easiest and most common method of incorporating phytochemicals into polymer films. In this approach, the bioactive compound, e.g., polyphenol extract, essential oil, or curcumin, is dissolved or dispersed in a compatible solvent and mixed with the polymer solution before film formation via solvent casting [82,83]. The mixture is then spread onto a flat surface and the solvent is evaporated under controlled conditions with the phytochemical evenly distributed throughout the polymer matrix. Solvent casting has many advantages like ease of implementation, compatibility with heat-sensitive compounds and the ability to produce transparent and homogeneous films at the laboratory scale [82,83]. However, the scalability and industrial applicability of this method are limited as the solvent removal process is energy intensive and residual solvent traces can still be detected in the final film [84,85]. Furthermore, direct blending usually leads to fast and uncontrolled phytochemical release due to their surface localization and poor interaction with the polymer matrix, which may cause their early depletion and reduced long-term efficiency [86,87].

Melt extrusion and thermoforming are scalable alternatives to solvent casting for thermoplastic biopolymers like PLA, PHBV and PBAT. The processes include compounding of the phytochemicals, mainly thermostable essential oils and polyphenols, with the polymer melt at controlled temperature and shear conditions [88,89]. However, heat sensitive bioactives can be degraded at high processing temperatures (150–200 °C for PLA and PHBV), and protective carriers (nanoparticles and cyclodextrins) or thermally stable compounds should be selected.

### 5.2. Encapsulation Systems

Encapsulation strategies have been developed as an effective method to overcome the disadvantages of direct blending, providing better stability, controlled release, and protection of phytochemicals from degradation during processing and storage [90,91]. Encapsulation systems used are nanoparticles, cyclodextrin inclusion complexes, liposomes, niosomes and emulsions, each with different advantages depending on the bioactive and polymer matrix. Nanoparticle encapsulation consists of the entrapment of phytochemicals into nanocarriers based on biocompatible polymers (chitosan, alginate, and PLGA), lipids, or inorganic materials (silica and metal oxides) [92,93]. Nanoparticles have high surface to volume ratios, tunable surface chemistry and can protect encapsulated substances from oxidation, hydrolysis and volatilization. Starch and gelatin films loaded with chitosan nanoparticles containing gallic acid or quercetin released sustained antioxidant activity and showed better antimicrobial activity than free phytochemicals. Similarly, silica nanoparticles functionalized with essential oil showed enhanced thermal stability and controlled release in PLA matrices [25,94,95].

Another popular encapsulation technique is cyclodextrin (CD) inclusion complexes, especially for hydrophobic phytochemicals such as essential oils, curcumin and tocopherols [90,91]. Cyclodextrins are cyclic oligosaccharides with hydrophobic cavities and hydrophilic exteriors, which form host–guest complexes with bioactive molecules, increasing their water solubility, thermal stability and resistance to oxidation. Essential oils (clove, thyme, and cinnamon) and polyphenols have been widely encapsulated with B-cyclodextrin and its derivatives (hydroxypropyl-β-CD and methyl-β-CD) to achieve homogeneous dispersion in hydrophilic polymer matrices and to control release kinetics through diffusion-controlled mechanisms.

Liposomes and niosomes are vesicular systems composed of phospholipids or non-ionic surfactants, respectively, and are biocompatible carriers for hydrophilic and lipophilic phytochemicals [96]. These systems protect bioactives from degradation, improve their bioavailability and allow sustained release triggered by environmental stimuli (pH, temperature, and enzymes). Liposome-encapsulated essential oils and polyphenols have been incorporated in chitosan, alginate, and gelatin films which show enhanced antimicrobial efficacy and increased shelf life for meat and dairy products.

Emulsion-based systems, such as traditional oil-in-water (O/W) emulsions and solid particle-stabilized Pickering emulsions (nanocellulose, protein nanoparticles, and clay) offer flexible approaches for incorporating hydrophobic phytochemicals into hydrophilic biopolymer matrices [97,98]. Pickering emulsions, in particular, have received much attention due to their excellent stability, absence of synthetic surfactants and multifunctional properties. It has been reported that starch, alginate and soy protein films incorporated with Pickering emulsions stabilized by nanocellulose or chitosan nanoparticles loaded with essential oils possess enhanced antimicrobial, antioxidant and water-vapor barrier properties when compared to films with free essential oils [99,100].

### 5.3. Electrospinning and Nanofiber Systems

Electrospinning has been a versatile method for the production of nanofibrous membranes with high surface-to-volume ratios, porosity, and tunable morphology for active and intelligent packaging applications [101]. In this process, a polymer solution containing phytochemicals is exposed to a high-voltage electric field, leading to the formation of continuous fibers with diameters ranging from tens of nanometers to several micrometers [102]. Electrospun nanofibers have several advantages over conventional films such as higher encapsulation efficiency, controlled release kinetics and better mechanical properties due to fiber entanglement [101,103]. Polyphenol-loaded electrospun fibers exhibited improved antioxidant and antimicrobial activity. The addition of anthocyanins, flavonoids, phenolic acids and plant extracts has reduced the oxidation, prevented microbial growth and maintained the sensory properties of fruits, vegetables, meat and seafood [101,102,104]. Fresh produce showed sustained antimicrobial release and extended shelf life with gelatin-based fibers containing bay laurel leaf essential oil, prepared using centrifugal spinning (a scalable version of electrospinning) [105]. Advanced spinning technologies including coaxial electrospinning, solution blow spinning, and microfluidic spinning allow for the production of core–shell fibers, Janus fibers, and hierarchical structures, further enhancing controlled release and multifunctionality [103,106]. In particular, coaxial electrospinning allows the encapsulation of bioactives in the core of the fiber, protecting them from environmental degradation and enabling stimuli-responsive release.

### 5.4. Hybrid Polyphenol-Nanoparticle Systems

**Figure 1:**
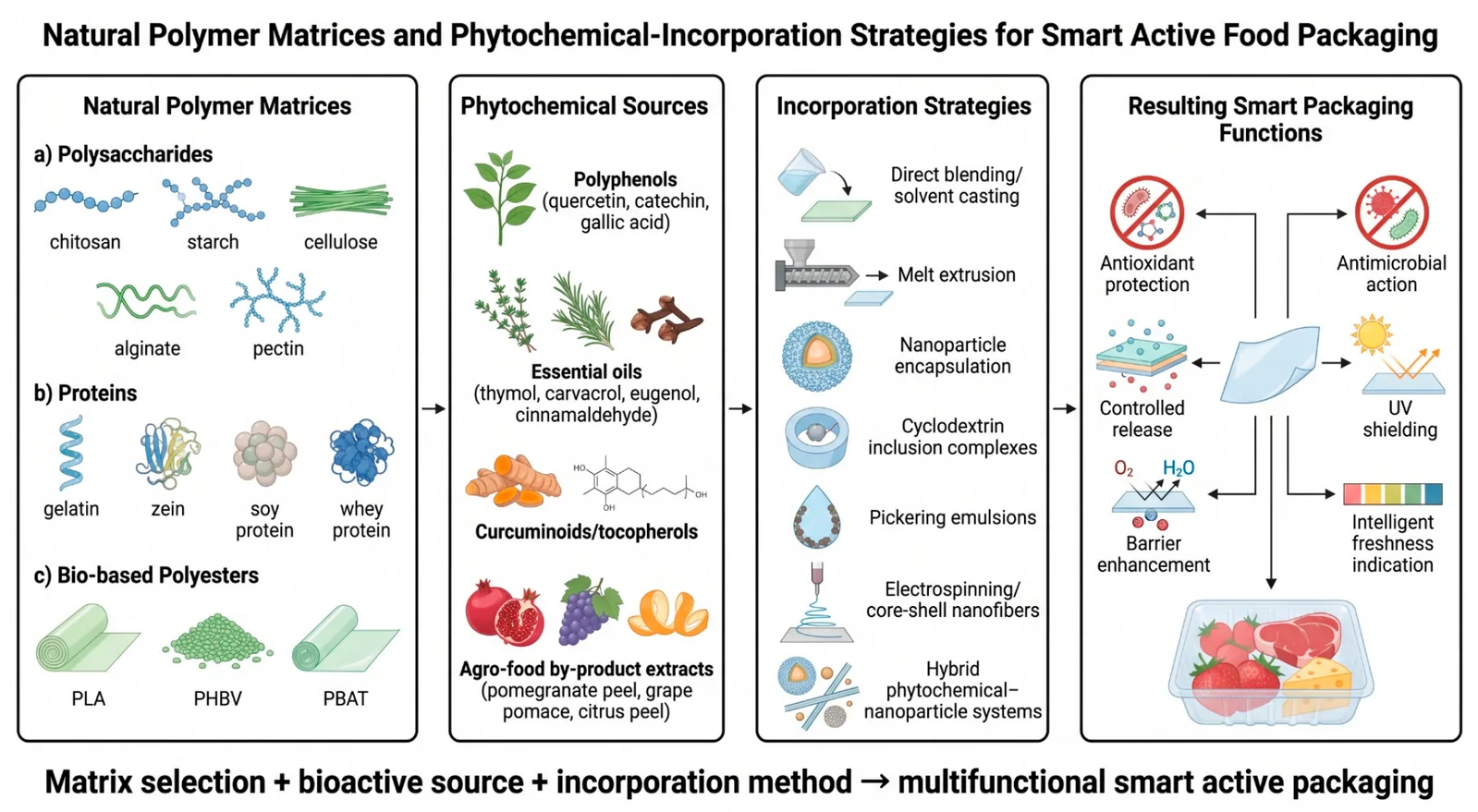
Natural polymer matrices and phytochemical-incorporation strategies for smart active food packaging. Polysaccharides, proteins, and bio-based polyesters can be combined with plant-derived polyphenols, essential oils, curcuminoids, tocopherols, and agro-food by-product extracts. Direct blending, melt processing, encapsulation, cyclodextrin inclusion, Pickering emulsification, electrospinning, and hybrid nanoparticle approaches modulate the distribution, stability, and release of phytochemicals within the polymer matrix. The resulting films can provide antioxidant, antimicrobial, UV-shielding, barrier-enhancing, controlled-release, and intelligent freshness-monitoring functions. (Created in BioRender. Raman, S. (2026) https://BioRender.com/j80djyf).

New developments in active packaging are hybrid systems of phytochemicals with inorganic or metallic nanoparticles, which exhibit synergistic effects for better antioxidant, antimicrobial and barrier properties [107,108,109]. Metal nanoparticles, such as Ag, and metal oxide nanoparticles, such as ZnO, TiO_2_, and CuO, can contribute antimicrobial activity and, depending on their composition, UV-blocking functionality, while phytochemicals provide complementary antioxidant and antimicrobial effects. For instance, ZnO NPs functionalized with gallic acid or quercetin showed improved radical scavenging activity and wider antimicrobial spectra than either component alone due to the synergistic ROS generation and disruption of membrane mechanisms [107,110]. Likewise, polyphenol extract (e.g., green tea and pomegranate peel)-stabilized Ag nanoparticles demonstrate enhanced colloidal stability, decreased cytotoxicity, and prolonged antimicrobial release in chitosan and PLA films [108,109]. Hybrid systems also allow multifunctionality beyond antimicrobial and antioxidant activity. For example, TiO2 nanoparticles and anthocyanins or curcumin have been used to develop pH-sensitive, color-changing films for intelligent packaging applications, where the color changes indicate food spoilage or pH changes [111,112]. These hybrid approaches exemplify the potential of the integration of phytochemicals with nanotechnology to develop next-generation smart packaging materials with tailor-made, multifunctional performance. The major natural polymer matrices, phytochemical sources, and incorporation strategies used to fabricate multifunctional smart active packaging films are summarized in Figure 1.

## 6. Release Kinetics and Stimuli-Responsive Designs

The efficacy of active packaging based on phytochemicals not only depends on the intrinsic bioactivity of the incorporated compounds, but also on their release kinetics from the polymer matrix into the food headspace or surface layer. Optimal release profiles should ensure that antioxidant and antimicrobial activities are maintained throughout the product’s shelf life, avoiding premature depletion or excessive migration that could affect sensory properties or breach regulatory limits [75,113]. This section discusses the basic mechanisms for the release of bioactives from polymer films, the mathematical models used to describe the release kinetics, and emerging stimuli-responsive designs that allow on-demand synchronized delivery of phytochemicals in response to food spoilage indicators.

### 6.1. Fundamental Release Mechanisms

The release of bioactives from polymer films involves diffusion, polymer relaxation, swelling and in some cases, matrix erosion or degradation [75]. The prevailing mechanism is governed by the physicochemical properties of the bioactive (molecular size, polarity, and volatility), the polymer matrix (hydrophilicity, crystallinity, and crosslink density), and environmental conditions (temperature, humidity, and pH) [114]. The most common release mechanism is Fickian diffusion, which is the transport of bioactive molecules from regions of high concentration inside the film to lower concentration in the surrounding environment along a concentration gradient [115]. The mechanism is predominant when the diffusion rate of the bioactive is much lower than the polymer relaxation rate, a situation often met in glassy or semi-crystalline polymers with limited mobility of the chains. For example, the release of polyphenols from chitosan films with natural extracts has been reported to follow quasi-Fickian diffusion behavior, suggesting that diffusion through the hydrated polymer network is the main release pathway [114].

Non-Fickian or anomalous transport is observed when diffusion and polymer relaxation occur at similar rates, leading to complex release profiles that are not classical Fickian behavior [75,116]. This mechanism is typical of hydrophilic, swellable biopolymers like gelatin, alginate and starch, where water uptake leads to chain relaxation and network expansion allowing for bioactive migration [117]. In Case II transport, a type of non-Fickian behavior, there is a sharp moving front between swollen and glassy regions, leading to zero-order (constant rate) release kinetics [116]. In highly hydrophilic or enzymatically degradable matrices, swelling-controlled release and erosion-mediated release are the main mechanisms. Water absorption leads to a large expansion of the network or partial dissolution, and an increased diffusion rate of the bioactive [118]. These mechanisms are frequently coupled with pH-triggered solubilization in edible films and coatings, particularly for ionizable phytochemicals such as polyphenols and essential oil components.

### 6.2. Mathematical Modelling of Release Kinetics

Mathematical models help quantify phytochemical release from polymer films and distinguish release profiles associated with diffusion, polymer relaxation, and other transport processes. However, the models differ in their physical basis: some describe transport under specified assumptions, whereas others provide empirical or semi-empirical representations of experimental curves. Model selection should therefore consider film geometry, swelling or erosion, release-medium conditions, and the fraction of the release curve being analyzed, rather than relying solely on goodness of fit. In the equations below, M_t_ denotes the cumulative amount released at time t, M_∞_ denotes the amount released at equilibrium under the specified experimental conditions, and *F*(*t*) = *Mt*/*M∞.* Unless otherwise stated, the expressions assume no initial burst or lag time [119,120].

#### 6.2.1. Zero-Order and First-Order Models

The zero-order model describes a constant release rate over the fitted interval:

*F*(*t*) = *k*_0_*t*

where *k*_0_ is the fractional release-rate constant. A zero-order profile can occur when the driving force and transport resistance remain approximately constant, or when coupled processes compensate for depletion. However, linear cumulative release does not uniquely identify diffusion, swelling, or erosion as the governing mechanism. The model is therefore useful for describing sustained delivery but should not be interpreted as mechanistic proof [120].

The first-order model describes release at a rate proportional to the remaining releasable amount:

*F*(*t*) = 1 − *exp*(−*k*_1_*t*)

#### 6.2.2. Fickian Diffusion from a Planar Film

A diffusion-based description is appropriate when transport is driven primarily by concentration gradients and the film retains approximately constant dimensions and diffusivity. For an initially uniform planar film of total thickness *L*, exposed on both faces to perfect-sink conditions, the solution of Fick’s second law is as follows:

$$F(t)=1-8/\pi^{2}\sum_{j=0}^{\infty }\frac{1}{{\left(2j+1\right)}^{2}}\exp\left[-\frac{{\left(2j+1\right)}^{2}\pi^{2}D\;t}{L^{2}}\right]$$

where *D* is the diffusion coefficient. This model explicitly links release to film thickness and molecular diffusivity. Its assumptions include one-dimensional transport, negligible edge effects, constant *D*, and negligible swelling, erosion, or external mass-transfer resistance. Films in contact with finite food volumes, one-sided exposure, or appreciable film–food partitioning require the corresponding boundary conditions rather than direct application of the perfect-sink expression [119].

#### 6.2.3. Higuchi Model

The Higuchi model describes a square-root-of-time release profile:

$$F(t)=k_{H}t^{\frac{1}{2}}$$

where *k_H_* is the normalized Higuchi release constant. The classical planar Higuchi model represents diffusion-controlled release from a matrix containing initially dispersed active material at a concentration exceeding its solubility in the matrix. Its assumptions include constant diffusivity, negligible matrix swelling and dissolution, one-dimensional transport, and sink conditions. Although square-root-of-time behavior is consistent with diffusion-controlled release in appropriate systems, this functional dependence alone does not establish that all classical Higuchi assumptions are satisfied. Its application to swellable or erodible biopolymer films therefore requires particular care [121].

#### 6.2.4. Korsmeyer–Peppas Model

The Korsmeyer–Peppas model is a semi-empirical power-law expression:

*F*(*t*) = *k_KP_t^n^*

where *k_KP_* is a kinetic constant and *n* is the release exponent. The exponent provides an indication of transport behavior only when interpreted with the appropriate geometry and fitting range. For an ideal planar slab, *n* = 0.5 is associated with Fickian diffusion, 0.5 < *n* < 1 with anomalous transport involving coupled diffusion and polymer relaxation, and *n* = 1 with Case II transport under the model’s applicable conditions. Different threshold values apply to cylindrical and spherical systems. The model is generally applied to the initial portion of release, commonly up to approximately 60%, rather than to the entire curve. Values outside the expected ranges require examination of burst release, geometry, heterogeneity, and model suitability rather than automatic mechanistic classification [120,122].

#### 6.2.5. Peppas–Sahlin Model

The Peppas–Sahlin model separates diffusion-associated and relaxation-associated contributions:

*F*(*t*) = *k_d_t^m^* + *k_r_t*^2^*^m^*

where *k_d_* and *k_r_* represent the diffusion-associated and relaxation-associated coefficients, respectively, and mmm is the Fickian exponent appropriate to the geometry. The first term describes the diffusion contribution, while the second represents the additional contribution associated with polymer relaxation. The model is useful for swellable matrices in which water uptake and chain rearrangement influence phytochemical transport. However, the fitted terms should be interpreted cautiously when coefficients are physically implausible or the assumptions and fitting interval are inappropriate [122].

#### 6.2.6. Weibull Model

The Weibull equation provides a flexible empirical description of release:

$$F\left(t\right)=1-\exp\left[-{\left(\frac{t}{\tau }\right)}^{\beta }\right]$$

where *τ* is a characteristic time and *β* is a shape parameter. Values of *β* < 1 describe a strongly decelerating release profile, *β* = 1 gives an exponential profile, and *β* >1 produces a sigmoidal profile with an initial accelerating phase. This flexibility makes the model useful for comparing release curves with different shapes. Nevertheless, its parameters do not uniquely identify diffusion, swelling, relaxation, or erosion; independent evidence is required for mechanistic interpretation [120,123].

#### 6.2.7. Model Selection and Interpretation

A single equation cannot describe all phytochemical–polymer systems. Candidate models should be selected according to the material, geometry, experimental boundary conditions and the desired fitting range. The model comparison should not be based only on the coefficient of determination but also on the residuals patterns, the plausibility of the estimated parameters, and the criteria penalizing for the number of fitted parameters. Also, comparisons should be based on the same response scales and the same fitting intervals. Mechanistic conclusions should be supported by independent measurements, e.g., swelling, mass loss, film-thickness changes or release under different environmental conditions. In active food packaging, partitioning into the food or headspace and interactions with food components further constrain the transfer of model parameters from food simulants to real products [119,120].

The fitted release parameters should be interpreted as properties of the complete matrix–phytochemical–medium system. Increasing crosslink density can restrict molecular transport by reducing network mesh size, although its effect depends on phytochemical dimensions and polymer–solute interactions [124]. Changes in matrix structure or solvent affinity can alter release rates without necessarily producing a corresponding change in *n*, which describes release-curve scaling rather than an intrinsic material property [119]. In the fractional Korsmeyer–Peppas expression, *k* has units of time^−*n*^; consequently, a universal numerical range is not meaningful without specifying time units, normalization, geometry, fitting interval, and experimental conditions. Simulant results also require food-specific validation: gelatin and chitosan films demonstrate that medium water activity, viscosity, and penetration into the film can alter release and partitioning [75].

### 6.3. Stimuli-Responsive and Smart Release Systems

**Figure 2:**
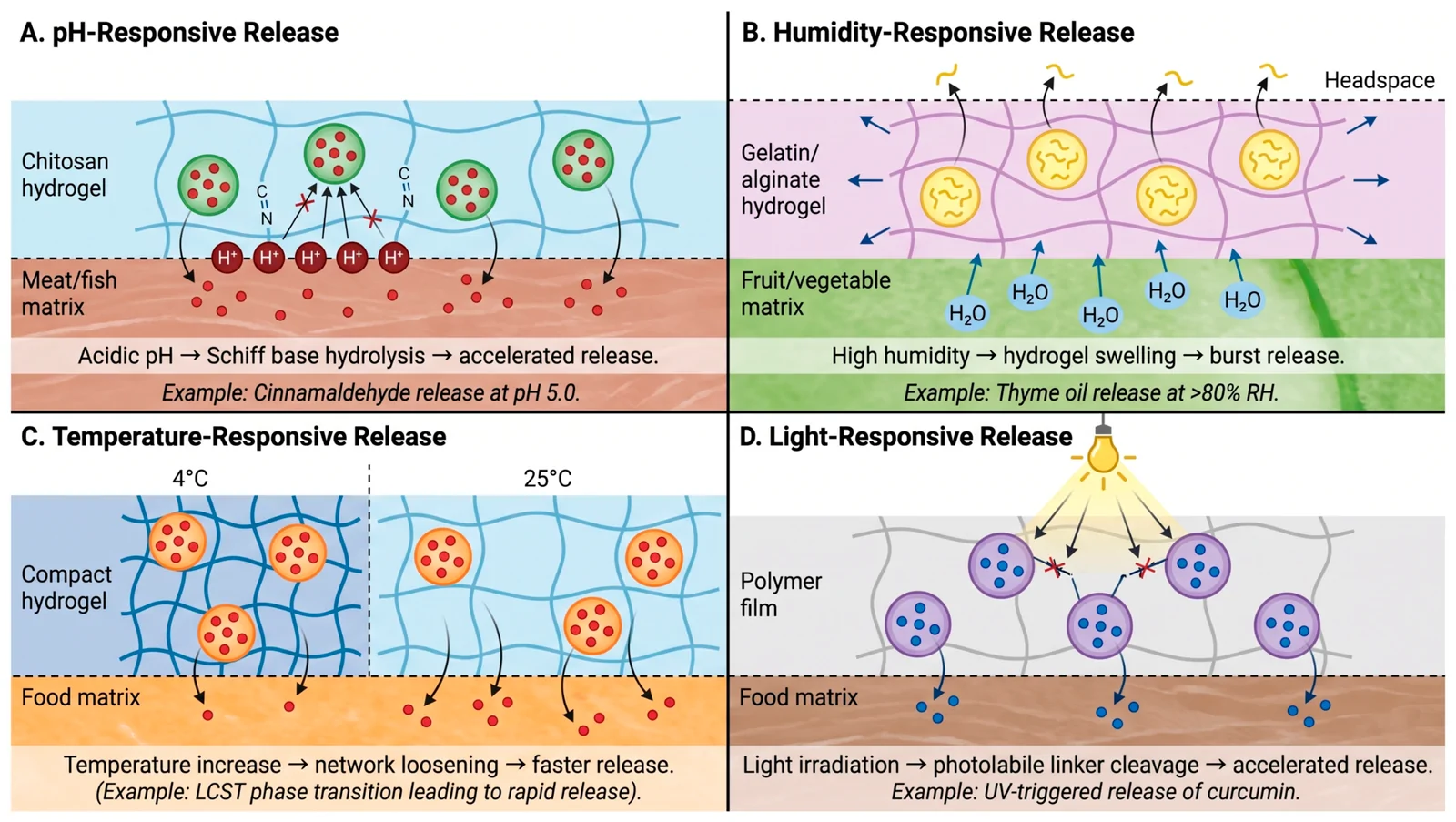
Stimuli-responsive release mechanisms in smart phytochemical-functionalized polymer packaging films. (**A**) pH-responsive release, where acidic conditions promote hydrolysis of Schiff-base linkages within a chitosan hydrogel and accelerate the migration of encapsulated phytochemicals into the food matrix. (**B**) Humidity-responsive release, where water-vapor uptake swells gelatin- or alginate-based hydrogel networks, increases mesh size, and promotes essential-oil release into the package headspace. (**C**) Temperature-responsive release, where elevated temperature induces network loosening or a lower critical solution temperature (LCST)-related phase transition, resulting in faster release of active compounds. (**D**) Schematic photocleavage-mediated release, in which irradiation cleaves photolabile groups in a suitably designed carrier or polymer network and facilitates bioactive release; this process is distinct from photodynamic antimicrobial activation. These mechanisms allow controlled delivery of antioxidants and antimicrobials according to changes in the packaging environment. (Created in BioRender. Raman, S. (2026) https://BioRender.com/j80djyf).

In general, active packaging systems release bioactives at a constant or diffusion-controlled rate, irrespective of the actual spoilage status of the packaged food. In contrast, stimuli-responsive or smart packaging systems are designed to alter release in response to specific environmental signals linked to food-quality deterioration, such as shifts in pH, increases in humidity, temperature abuse, or the presence of spoilage metabolites, including volatile amines, organic acids, and ethylene [19,125]. The release of incorporated bioactives can be changed by changes in pH, and therefore by modifying the ionization of polymer functional groups, the swelling or solubility of the matrix or the stability of acid-labile bonds. An acid-responsive system based on dynamic Schiff-base linkages is shown in Figure 2A. In properly designed chitosan-based networks, acid-catalyzed hydrolysis of these linkages can decrease network connectivity and promote the release of entrapped substances. If the bioactive that contains the aldehyde, for example cinnamaldehyde, is attached to chitosan by an imine bond, then hydrolysis can directly release the bound compound. It is important to distinguish these two release configurations. Chitosan–cinnamaldehyde systems exemplify the importance of Schiff-base chemistry in pH-responsive antimicrobial delivery for food preservation. However, the direction and magnitude of the response is polymer and bond chemistry dependent and acidic conditions should not be portrayed as a universal trigger for all pH responsive packaging. Representative stimuli-responsive release mechanisms relevant to phytochemical-functionalized polymer packaging films are illustrated in Figure 2.

The panels present generalized mechanistic schematics rather than exact representations of individual experimental formulations. Related primary studies have demonstrated acid-responsive aldehyde release from chitosan Schiff bases [126], humidity-dependent release from CMCS/gelatin hydrogel pads [127], and thermoresponsive release from PNIPAAm-covered PLA nanofibers [128] in food-preservation applications. Photocleavage-mediated curcumin release has been demonstrated in polymeric micelles [129]; its extension to a food-packaging film in Panel D is conceptual. Trigger conditions depend on the specific formulation and experimental environment and should not be interpreted as universal thresholds.

Humidity-sensitive systems exploit the increase in relative humidity (RH) that is typically associated with microbial spoilage and enzymatic browning in fresh produce prior to or during storage [125,130]. Hydrophilic biopolymer networks, such as chitosan, alginate and gelatin hydrogels, can absorb water vapor and swell, leading to an increase in the mesh size and diffusivity, and thus accelerating the release of encapsulated bioactives [125,130]. Humidity-responsive hydrogels incorporated with microcapsules containing essential oils are promising for moisture-sensitive foods, as they can obtain burst release upon RH increase, effectively inhibiting mold growth and enzymatic browning.

Temperature-responsive systems tackle the important problem of temperature abuse in cold chain logistics, which accelerates microbial growth and lipid oxidation [128,131]. Poly (N-isopropylacrylamide) (PNIPAM) and its copolymers are examples of thermoresponsive polymers that display reversible volume phase transitions around the lower critical solution temperatures (LCSTs) and control the release rates of bioactive compounds in response to temperature changes [128,131]. Thermoresponsive hydrogels and microcapsules have been studied for on-demand antimicrobial release in response to storage temperatures above safe thresholds, but their use in food packaging is less common due to regulatory and safety issues.

Light-triggered release. If the carrier contains photoresponsive groups which are cleaved or otherwise structurally altered upon irradiation, light can be used to control bioactive delivery. Figure 2D illustrates schematically photocleavage-mediated release: irradiation at an appropriate wavelength cleaves photolabile groups in a carrier or network, altering its integrity or permeability and enabling bioactive release. For instance, light-induced delivery of curcumin using photocleavable polymeric micelles has been investigated in antimicrobial photodynamic applications. Such delivery-system evidence provides a mechanistic basis for the schematic, but should not be considered as direct validation of a food-contact packaging film. Photocleavage-mediated delivery should also be contrasted with curcumin-mediated photodynamic inactivation, where illumination activates a photosensitizer to produce antimicrobial reactive oxygen species without necessarily enhancing its release. Curcumin-mediated photodynamic activity has been shown in food-packaging studies, but the release mechanism and its sensitivity to illumination should be evaluated separately.

A new frontier in intelligent packaging is spoilage metabolite-responsive systems in which release is triggered by specific chemical signatures of food deterioration such as volatile basic nitrogen (TVB-N) from protein degradation, ethylene from fruit ripening or biogenic amines from microbial activity [132,133]. For instance, pH-sensitive films of anthocyanin or curcumin loaded with ammonia or amine vapors change color as a visual indication of spoilage and release antimicrobial essential oils to reduce further degradation [133].

### 6.4. Synchronizing Release with Food Oxidation Kinetics

One of the main challenges in the design of active packaging is to match the release kinetics of antioxidants and antimicrobials with the actual deterioration rates of certain foods. For lipid-rich foods, such as meat, fish, and oils, antioxidant release should be designed to suppress lipid peroxidation throughout storage, whereas antimicrobial delivery should be matched to the target microorganisms, food matrix, and intended storage conditions [12,20,134]. Recent developments in multi-stimuli-responsive systems are designed to tackle this problem by incorporating multiple triggers (pH, temperature, and humidity) into a single platform that allows adaptive release profiles that can dynamically respond to changing spoilage conditions.

Food packaging research has revealed direct evidence of stimulus-dependent delivery in certain food–material combinations. For example, pH-sensitive packaging has been studied for pork and thermo-responsive nanofibers for blackberry. They need to be discriminated from evidence for quantitated release in parallel with food spoilage. Quantifying release independent of microbial growth or oxidation in the same storage conditions is not equivalent to feedback-controlled delivery; better preservation is not feedback-controlled release.

Future packaging is informed by mechanistic design studies on dynamic-covalent hydrogels for biomaterial delivery, antibacterial activity, or wound healing. These studies are discussed in terms of material research, not demonstrated applications for food packaging. Biocompatibility and/or antimicrobial efficacy relevant to their use as food-packaging materials is not the same as food-contact safety, migration performances, sensory acceptability, or shelf-life extension. This would require food-specific leaching tests for safety and appropriate migration/shelf-life studies. Therefore, such hydrogels are anticipated to be used in food packaging in the future [20,135,136,137].

## 7. Functional Performance: Antioxidant, Antimicrobial, Barrier, and Mechanical Properties

In the end, the practical utility of the phytochemical-functionalized active packaging films is determined by their capacity to offer demonstrable enhancements regarding food preservation, while preserving or upgrading the physical integrity of the packaging material itself. This section discusses the impact of incorporation strategies, polymer matrix selection and phytochemical loading on four key performance dimensions: antioxidant capacity, antimicrobial efficacy, barrier properties (oxygen, water vapor, and UV) and mechanical behavior (tensile strength, elongation, and puncture resistance). Knowledge of these structure–function relationships is required to optimize film formulations for particular food uses.

### 7.1. Antioxidant Capacity and in Vitro Assays

Antioxidant activity is usually assessed through standard in vitro radical-scavenging assays such as DPPH (2,2-diphenyl-1-picrylhydrazyl), ABTS (2,2′-azino-bis (3-ethylbenzothiazoline-6-sulfonic acid)), FRAP (ferric reducing antioxidant power) and ORAC (oxygen radical absorbance capacity) [138]. These assays test the capability of phytochemical-loaded films to transfer hydrogen atoms or electrons to stable radicals or oxidized metal complexes as a surrogate of the intrinsic reactivity. Films enriched with extracts containing high levels of polyphenols (green tea, grape seed, and pomegranate peel) or essential oils (clove, thyme, and cinnamon) exhibit high DPPH and ABTS radical scavenging activities, often with inhibition values higher than 50–80% at phytochemical loadings of 1–5% (*w*/*w*) [109]. For example, chitosan–starch films containing 0.45% of Portulaca oleracea extract led to a 28.7% reduction in TBARS (thiobarbituric acid reactive substances) in pork meat during 16 days of refrigerated storage, which was consistent with strong in vitro DPPH activity [139]. Similarly, PLA films with 2.5% pomegranate peel extract reduced the formation of TBARS by 50% in chicken jerky after 9 days due to the high phenolic content and radical-scavenging capacity of the extract.

However, in vitro antioxidant assays do not necessarily correlate with in-food performance, as efficacy can be modulated by bioactive stability, release kinetics, food matrix interactions, and competing reactions. For instance, anthocyanin-loaded films may exhibit high DPPH activity in acidic buffers, but lose activity in neutral pH foods due to structural degradation [140]. Therefore, further evaluations such as TBARS for lipid peroxidation, carbonyl content for protein oxidation and colorimetric analysis for pigment stability are necessary to confirm the antioxidant activity in real food systems.

### 7.2. Antimicrobial Efficacy and Mechanisms

Antimicrobial activity is evaluated with standardized methods such as agar disk/well diffusion, broth microdilution (minimum inhibitory concentration, MIC), time–kill kinetics, and biofilm inhibition/eradication assays [141,142]. In general, the essential oils and the films loaded with polyphenols show broad-spectrum activity against Gram-positive bacteria (Staphylococcus aureus, Listeria monocytogenes, and Bacillus cereus), Gram-negative bacteria (Escherichia coli, Salmonella Enteritidis, and Pseudomonas aeruginosa), yeasts (Saccharomyces cerevisiae), and molds (Aspergillus, Penicillium, and Botrytis) [143].

Essential oils rich in phenolics (clove, thyme, oregano, and cinnamon) tend to show the most significant antimicrobial effects, with MIC values ranging from 0.1 to 1.0 mg/mL against common foodborne pathogens. For example, chitosan films loaded with cinnamon oil inhibited the biofilm formation of Salmonella Enteritidis by 50–65% at MIC, while the preformed biofilms were reduced by <40%, indicating that prevention is more effective than eradication [141]. In a similar study, the nanoemulsions of oregano oil incorporated into hydroxypropyl methylcellulose films demonstrated improved antimicrobial activity against free oil, which was attributed to improved dispersion and a sustained release [144].

Mechanistically, antimicrobial action is due to membrane disruption, enzyme inhibition, interference with efflux pumps and blockade of quorum sensing, often synergistically overcoming microbial resistance [145,146]. Synergistic effects have been observed for essential oils with metal and metal oxide nanoparticles (Ag, ZnO, and CuO) in hybrid systems and ZnO-gallic acid and Ag-polyphenol composites have exhibited broader spectra and lower MICs compared to the individual components [143,147].

### 7.3. Barrier Properties: Oxygen, Water-Vapor, and UV Protection

Barrier performance is a key factor in the functionality of packaging, and it impacts the shelf life, weight loss, and oxidative stability of packaged foods. The main parameters are the oxygen permeability (OP) and water-vapor permeability (WVP), and the lower the values, the better the barrier properties [148]. The phytochemical incorporation can enhance or decrease barrier properties depending on the polarity of the compound, loading level and interaction with the polymer matrix [149]. Hydrophobic essential oils and lipophilic polyphenols (curcumin and tocopherols) are frequently employed to lower WVP by filling the free volume and increasing hydrophobicity, especially in hydrophilic biopolymers such as chitosan, starch and gelatin [150]. For example, cassava starch films with essential oils showed lower WVP and better water resistance but lower tensile strength [148]. Conversely, high loadings (>5%) of hydrophilic polyphenols could increase WVP by disturbing the packing of polymer chains and creating diffusion channels [149].

The addition of phytochemicals generally improves oxygen barrier properties, particularly when combined with nanofillers (nanocellulose, clay, and metal oxides) that establish tortuous diffusion pathways [151]. Pectin-based coatings have been investigated as oxygen-barrier materials for food-packaging applications [152]. Another important characteristic is the UV blocking ability as UV radiation accelerates lipid oxidation and pigment degradation [153]. Polyphenols and essential oils with conjugated double bonds (cinnamaldehyde, curcumin, and anthocyanins) provide an intrinsic UV-shielding effect protecting light-sensitive foods through UV-vis light absorption.

### 7.4. Mechanical Properties: Tensile Strength, Elongation, and Puncture Resistance

Mechanical integrity means that packaging films can withstand forces related to handling, transport, and storage without tearing or rupturing. The important parameters are tensile strength (TS), elongation at break (EB) and puncture resistance, which are generally determined by universal testing machines according to ASTM or ISO standards. The incorporation of phytochemicals often involves trade-offs between mechanical strength and flexibility. Hydrophobic essential oils and lipophilic compounds often act as plasticizers, increasing EB but decreasing TS and Young’s modulus by disrupting intermolecular hydrogen bonding. Control films had lower elongation at break and higher tensile strength than hydroxypropyl methylcellulose films with oregano EO nanoemulsions [144]. Likewise, the incorporation of cinnamon oil and chitosan into yam starch films significantly decreased TS (from 20.3 MPa to 4.09–7.71 MPa) with increasing oil loading [154].

On the other side, nanocomposite reinforcement can make up for the mechanical losses. Nanocellulose, ZnO and CuO nanoparticles have been shown to improve TS and Young’s modulus in chitosan, starch and PVA films by forming percolating networks and enhancing interfacial interactions [144,155]. For instance, chitosan/hydroxypropyl cellulose films with CuO nanoparticles showed significant improvements in terms of TS and modulus compared to the controls, but excessive loading of nanoparticles (>2%) resulted in aggregation and property deterioration. Cross-linking strategies (chemical (genipin, citric acid, and Schiff base) or physical (heat and UV)) also improve mechanical stability by increasing the network density and reducing the chain mobility. For example, chitosan hydrogels crosslinked with Schiff base presented improved tensile strength, water resistance, and pH-responsive release [156].

### 7.5. Balancing Multifunctionality

To achieve optimal multifunctionality, a careful balance of phytochemical type, loading level and matrix composition is needed to avoid compromising one property for another. Hybrid systems combining encapsulation, nanocomposites and cross-linking are promising routes to simultaneously improve antioxidant, antimicrobial, barrier and mechanical performance [157,158,159]. Recent research reported a multi-cross-linked pectin film with high elongation (196%), low oxygen permeability, excellent TS, water-vapor resistance, antibacterial activity and oxidation resistance, demonstrating the feasibility of integrating multiple functionalities in a single platform [160]. The following section translates these functional properties into practical applications by presenting case studies from various food categories and summarizing the main results in tables.

**Figure 3:**
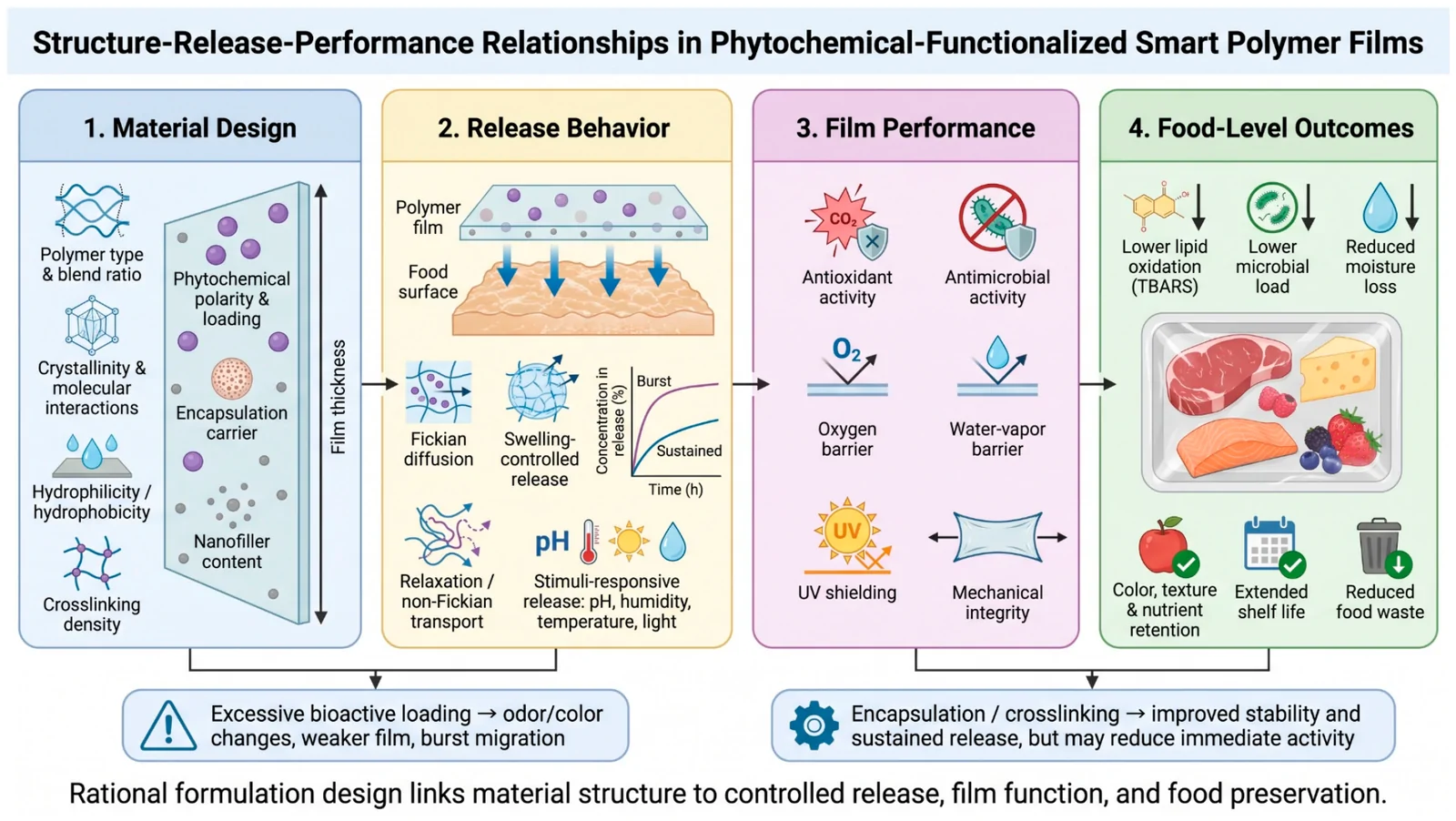
Structure–release–performance relationships governing phytochemical-functionalized smart polymer films. Polymer composition, physicochemical interactions, film thickness, cross-linking density, phytochemical loading, encapsulation strategy, and nanofiller incorporation determine the release mechanism and rate of active compounds. Release behavior subsequently influences antioxidant and antimicrobial effectiveness, barrier performance, UV shielding, and mechanical properties. Optimized design enables reduced lipid oxidation and microbial growth, preservation of sensory quality, shelf-life extension, and reduced food waste, whereas excessive loading or poorly controlled migration can cause sensory alteration, inadequate mechanical performance, or premature depletion of the active agent. (Created in BioRender. Raman, S. (2026) https://BioRender.com/j80djyf).

**Figure 4:**
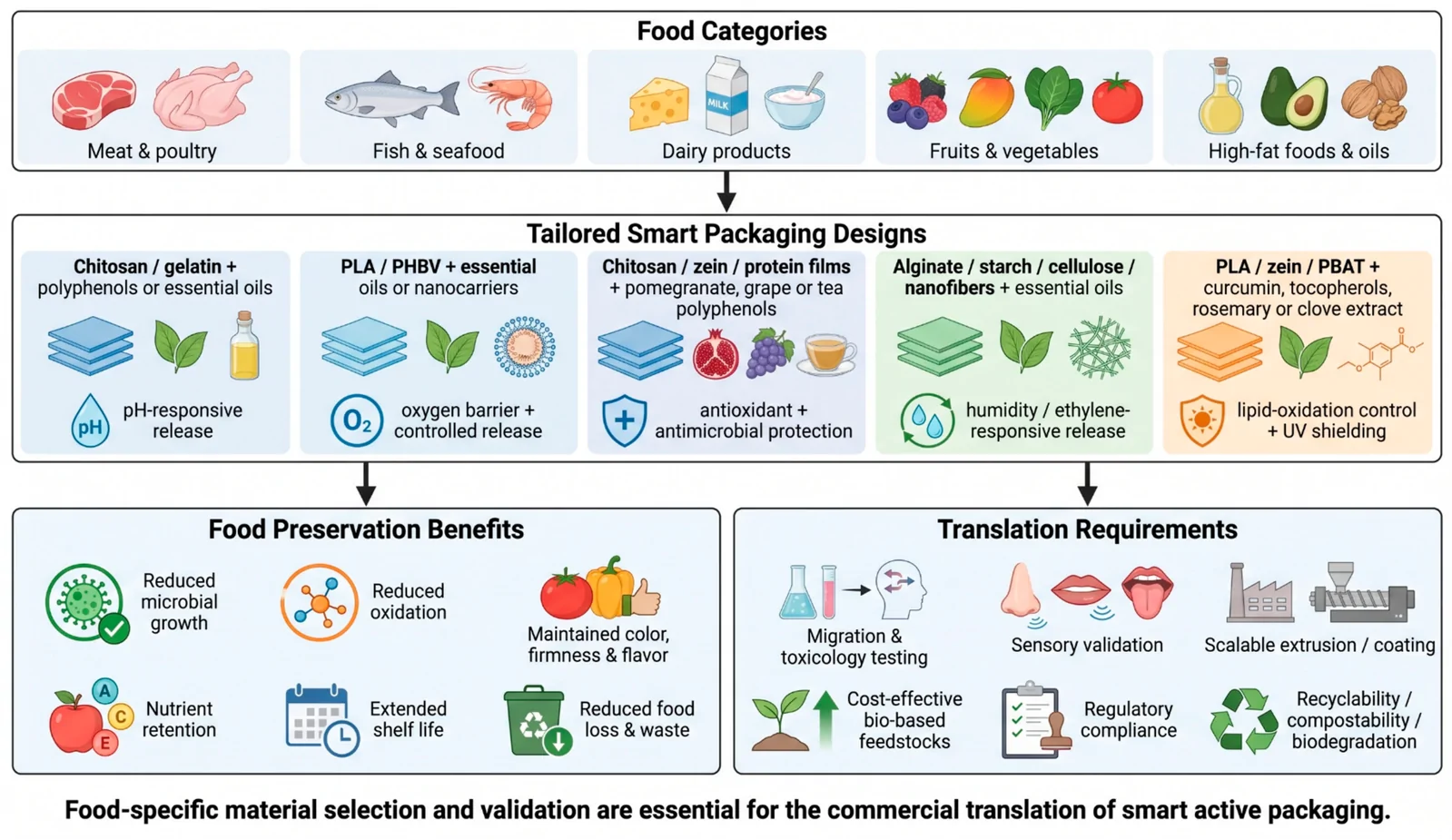
Food-specific applications and translational pathway for phytochemical-functionalized smart polymer packaging. Different foods require tailored combinations of polymer matrix, phytochemical additive, release mode, and barrier performance. Chitosan- and protein-based films are particularly relevant for meat and dairy products, biodegradable polyester systems are useful for fish and high-moisture foods, and polysaccharide- or nanofiber-based systems can support fresh-produce preservation. Successful translation requires demonstration of food-level efficacy, controlled migration, sensory acceptability, manufacturing scalability, regulatory compliance, and environmentally appropriate end-of-life management. (Created in BioRender. Raman, S. (2026) https://BioRender.com/j80djyf).

For practical formulation selection, the relationships discussed in this review can be organized into a qualitative decision sequence linking phytochemical structure, polymer matrix, incorporation method, release behavior, and food-level outcomes. Selection should begin with the dominant deterioration pathway and intended storage conditions of the target food, followed by identification of a phytochemical with suitable activity and processing stability. The matrix and incorporation method should then be assessed for their effects on retained active content, film morphology, thickness, swelling, and mechanical integrity. Release should be quantified under relevant contact conditions, with kinetic models selected according to film geometry, boundary conditions, and evidence of swelling or erosion rather than goodness of fit alone. Candidate formulations should subsequently be evaluated against appropriate controls using predefined food-level endpoints, such as microbial growth, lipid oxidation, and sensory acceptability, together with migration and handling requirements. A formulation should advance only when its delivery profile supports preservation without unacceptable sensory changes, migration, or loss of material integrity. Numerical targets must be established for the specific food–material–storage combination; this review does not derive universal parameter ranges or provide a validated predictive model. Accordingly, Figure 3 and Figure 4 should be interpreted as conceptual summaries supporting application-specific selection and validation. The interdependent relationships among polymer structure, phytochemical interactions, release behaviour, and packaging performance are summarized in Figure 3.

## 8. Applications in Real Food Systems

Overall packaging stability is a function of the intrinsic properties of the polymer matrix, phytochemicals incorporated within the matrix, and interaction of the matrix-phytochemical complex with the food environment. The matrix controls the transport of gases/moisture and the retention/release of active compounds, and released or surface-available phytochemicals may inhibit oxidation or microbial action. These contributions should be differentiated: protection of food by improved barrier properties of the film, release of antioxidants, antimicrobial activity, or combinations of different processes. The relative importance of these contributions will be dependent on storage conditions, food composition, dominant deterioration pathway, and concentration of active compounds on the food or in the package headspace.

The final proof of the concept of phytochemical-functionalized active packaging is its real storage conditions in different food matrices. Although in vitro assays provide useful information on intrinsic bioactivity, the complex nature of food systems such as interactions within the matrix, competing reactions, variations in the microenvironment, and sensory limitations could affect efficacy [64,65]. This section provides application-oriented case studies in meat and poultry, fish and seafood, dairy products and fresh produce and illustrates how laboratory results are translated into active films for the practical extension of shelf-life and preservation of quality. The key findings are summarized in Table 1 for easy cross-comparison and to facilitate formulation design for specific food categories.

Table 1 summarizes selected applications of phytochemicals as food-packaging agents, and is only a qualitative and not a comparative evaluation of preservation efficacy. Phytochemical formulations, loads, control treatment, packaging, food, storage, sampling time, and shelf-life definitions vary and complicate result interpretation. Observations of microbiological reduction and oxidation in foods should not be confused with in vitro antimicrobial or antioxidant activities. Similarly, a more extended storage experiment should not be interpreted as a shelf-life extension in the absence of a proper control and a defined endpoint for acceptability. Therefore, the reported applications should be interpreted taking into account the experimental conditions and should not be used to rank formulations across studies.

### 8.1. Meat and Poultry Products

Meat and poultry films functionalized with phytochemicals target oxidative spoilage and microbial growth on surfaces. Antioxidants reaching the interface of food and film may interfere with propagation of lipid radicals, and molecules with appropriate metal chelating groups may also reduce metal oxidation catalysis. Depending on the volatility of the antimicrobials and of the delivery system, the antimicrobial compounds are active in the headspace or at the product surface. Transfer of oxygen and moisture is also limited by the polymer matrix that modulates release of bioactives. A two-pronged approach or mechanism (not single) is therefore plausible to explain, for example, the chemical abnormality of a physical phenomenon, that of suppressed growth of microbes and otherwise reduced lipid oxidation (and vise versa). Such measurements should be considered in association with color, drip loss, and texture changes rather than as indicators of a given molecular pathway.

Meat and poultry are highly susceptible to microbial spoilage and lipid oxidation due to their high water activity, being rich in nutrients and heme iron content which catalyzes peroxidation reactions [65,165]. Chitosan-based coatings with tea polyphenols, anthocyanins and essential oils have shown excellent potential for prolonging the shelf life of chilled pork, beef and poultry under refrigerated storage conditions [65,161]. A recent study reported that chitosan coatings containing blackberry anthocyanins and tea polyphenols (CS/BA/TP) improved preservation performance compared with chitosan alone, with reported reductions in total viable counts (TVC), TBARS, and total volatile basic nitrogen (TVB-N) after 12 days of storage at 4 °C [161]. The combination treatment also retained color stability (higher L* and a* values) and reduced drip loss, which could be attributed to improved antioxidant protection and microbial inhibition. Similarly, the shelf life of plant-based meat products coated with 1% chitosan, 2.5% tea polyphenols, and 0.04% nisin was 56 days under refrigeration, and the microbial counts only reached the national standard limits at the endpoint [161,166].

Beef preserved with pomegranate peel extract (PPE) immobilized in chitosan/PEO nanofibers resulted in significant reductions in E. coli O157:H7 numbers (2.96 log CFU/g at 4 °C over 10 days and 5.80 log CFU/g at 25 °C over 7 days), indicating the capability of nanofibrous delivery systems for high-risk pathogens [167]. In bilayer PLA/chitosan–starch films, bitter orange essential oil (1.2% BOEO) was found to effectively inhibit spoilage bacteria (total bacterial count, psychrotrophs, lactic acid bacteria, and Enterobacteriaceae) in rainbow trout filets for 16 days at 4 °C, and to slow pH rise and TBA value increases [65].

### 8.2. Fish and Seafood

Seafood packaging must slow its spoilage by microbes and oxidation of sensitive lipids. Antioxidants can retard oxidation chain reactions, and antimicrobials can inhibit (susceptible) spoilage organisms at the food surface. A good barrier to oxygen can further limit oxygen delivery, but this depends on the overall package and storage conditions. As with release behavior, this may be particularly relevant since wet food–film interfaces may affect polymer hydration and bioactive compound transport. Antioxidant preservation correlates with lower leak–oxidation values, and delayed microbial spoilage correlates with lower counts and slower accumulation of volatile nitrogenous compounds. However, such a result provides no direct indication of cellular targets of the incorporated compounds.

Fish and seafood are even more perishable than meat due to the high content of unsaturated fatty acids which are prone to quick oxidation and the presence of native microflora including psychrotrophic spoilage organisms [65,165]. Active packaging strategies for fish are aimed at delaying lipid oxidation (TBARS, peroxide value, and K-values) and inhibiting microbial growth (TVC, psychrotrophs, Pseudomonas, and Shewanella). PLA-based three-layer films incorporating cinnamon essential oil (CEO) and graphene nanoplatelets (GNP) have been investigated for fish-ball packaging. However, the reported preservation outcomes should be interpreted within the experimental storage conditions and should not be equated with a validated safe shelf life without pathogen-relevant evidence. It has been reported that composite edible films containing perilla essential oil (6 μL/mL), glycerol monolaurate (18.4 μg/mL), chitosan (14.2 mg/mL), and nisin (11.0 μg/mL) preserved ready-to-eat fish balls during 12 days of storage [168]. The best formulation was chosen based on the antimicrobial activity against Staphylococcus aureus and produced lower total viable bacterial loads, total volatile basic nitrogen, and appearance deviation with fish balls compared with controls. However, results show improved preservation performance but not necessarily pathogen control or ambient shelf-life assessment [65].

### 8.3. Dairy Products

Coatings that are plastically molded or energetic can provide antimicrobial protection of the surface and regulation of moisture loss and oxidative damage, particularly for cheese and other dairy foods. The coating acts as a transport barrier, and phytochemicals added to the coating can contribute to additional activity if they are available to the microorganisms or migrate to the food-contact area. Protein films can provide good oxygen-barrier properties, although they can be affected by humidity and formulation. Retained moisture, for instance, should be discussed in terms of water transport; lower microbial counts should be discussed in terms of antimicrobial protection; and lower lipid-oxidation values should be discussed in terms of antioxidant activity. These functions may coincide, but without evidence they cannot be ascribed to the same mechanism.

Dairy products, especially soft cheeses with high moisture and low salt content, are easily contaminated by yeast, mold and bacteria, so they need effective antimicrobial and antioxidant protection [65]. Domiati cheese was coated with carboxymethyl chitosan nanoparticles loaded with pomegranate peel extract (CCS LP) as an edible coating, revealing a 66.17% reduction in the growth of Staphylococcus aureus at 800 μg/mL, while the moisture content was 61.02% after 28 days of refrigerated storage compared with 53.89% for uncoated controls [164]. Microbial loads were significantly suppressed with total bacterial counts and yeast/mold counts at 5.44 and 3.77 log CFU/g, respectively, as compared to 7.44 and 5.07 log CFU/g in uncoated cheese [164]. Zein coated films with pomegranate peel extract on Himalayan cheese (kalari) showed concentration-dependent antioxidant protection with the lowest oxidation levels at the highest levels of extract over a 30-day storage period [169]. The incorporation of chitosan and grape bagasse extract into BC films reduced the TBARS value from 0.42 to 0.35 mg MDA/kg after 60 days of storage, corresponding to an approximately 16.7% reduction in this indicator of lipid oxidation.

### 8.4. Fruits and Vegetables

In fresh produce, packaging preservation is a question of limiting gas exchange, moisture, and perhaps microorganism action. An appropriate film/coating adjusts oxygen and carbon dioxide exchange around the product and limits water loss and thus can influence respiration-related deterioration, weight loss, and texture retention. However, the gas permeability should be selected according to the commodity and storage condition, and a better gas barrier is not always good for a respiring product. Antimicrobials can provide some protection against surface microbes to which the product is subject, and antioxidation may help protect against oxidation quality losses. Delayed ripening or restricted browning should not be attributed to ethylene exclusion or direct inhibition of enzymes unless these mechanisms have been measured.

Active and intelligent packaging is a suitable candidate for fresh produce since it suffers post-harvest losses from respiration, ethylene production, microbial decay and enzymatic browning [64,65]. Chitosan-based coatings have been widely studied for bananas, strawberries, mangoes, and tomatoes and have shown delayed ripening, reduced weight loss, and maintenance of firmness and color [64,170]. Chitosan coatings (2%) modified with diepoxy-polyethylene glycol and trans-cinnamaldehyde maintained total soluble solids (TSS), titratable acidity (TA) and appearance of bananas for 12–24 days, significantly extending shelf life under ambient conditions [64]. Zhang et al. (2022) [171] further reported that chitosan + gum arabic coatings containing cinnamon oil (0.05–0.5%) delayed the loss of firmness, weight loss, acidity loss, and color change of bananas, and the best effect was observed at 0.25% CEO [64]. Starch/PBAT films containing microcapsules of cinnamon essential oil reduced strawberry weight loss by 49% and decay incidence from 56.5% to 26.3% after 6 days of storage [135]. The ZnO and Ag2O nanoparticles on ‘Cavendish’ bananas had better color parameters (L*, b*, a*, h°) and decreased microbial activity by 45% compared with the controls at the end of storage [171]. Under tropical conditions, 2% chitosan coatings on ‘Gran Enano’ bananas delayed visible ripening and browning, increasing shelf life from 13 to 17 days while preserving physicochemical and antioxidant quality [170].

### 8.5. Cross-Cutting Insights

Several trends are identified across the various food categories: (i) hydrophobic essential oils are very effective in high-moisture foods (meat, fish, and dairy) where their vapor-phase delivery enhances the antimicrobial efficiency; (ii) polyphenol-rich extracts are very effective in lipid-dense matrices where antioxidant preservation is a key requirement; (iii) nanofibrous and nanoparticle carriers improve the stability of bioactives and allow controlled release; and (iv) sensory qualities (odor, taste, and color) should be carefully traded off against functional benefits, especially in delicate products like dairy and fresh produce [25,64,65,166,172].

## 9. Challenges, Safety, and Regulatory Aspects

Laboratory and pilot studies have demonstrated promising functional performance of phytochemical-functionalized active packaging; however, there are several critical challenges to address for widespread commercialization. These challenges include safety and regulatory compliance, sensory and consumer acceptance, scalability and cost effectiveness, and environmental life-cycle considerations. In this section, each of these dimensions is considered, and a realistic view of barriers and opportunities for translation from bench to market is presented. The pathway from phytochemical selection and film design to food-specific validation, scale-up, regulatory assessment, and commercial implementation is summarized in Figure 4.

### 9.1. Regulatory Frameworks and Migration Limits

Active and intelligent packaging materials are subject to strict regulations to ensure that any substances transferred from the packaging to the food do not pose health hazards or cause unacceptable changes in the food’s composition, taste, or odor [171,173]. Active packaging in the European Union is primarily governed by Regulation (EC) No. 450/2009 on active and intelligent materials and articles intended to come into contact with food (which is parallel to the Framework Regulation (EC) No. 1935/2004 [174]. These regulations require that substances released from active packaging comply with the relevant EU food additives legislation and any intentional release must be technologically justified and safe for human consumption [175].

Regulatory compliance is the foundation of migration testing, with explicit requirements for food simulants, time and temperature conditions for both overall and specific migrations, particularly for plastics [176,177]. The overall migration limit (OML) is 10 mg/dm^2^ of the food-contact surface (or 60 mg/kg food) but specific migration limits (SMLs) apply to individual substances or classes of substances listed on the Union List [178]. Additional amendments have limited the use of bisphenol A (BPA) and related bisphenols in food-contact materials, such as Commission Regulation (EU) 2024/3190, in light of developing safety concerns [179,180].

Practical feasibility consists of practical issues relating to phytochemical release, activity on specific foods, and any regulatory constraints. For example, a study has demonstrated that treatment eliminating 3.2 ± 0.2 logs of Escherichia coli compared to an incubated control might be feasible since eugenol at 1.1 ± 0.5 mg/g was depth-migrated from PHBV bilayer films into fresh cheese after six days at 10 °C [181]. These results are effective treatment but not minimum effective dose or compliant with legislation. Displacement around the sample (incidental migration) should not arbitrarily impede deliberate active release as provided for in relevant food law and Article 9 of Regulation (EC) 450/2009 [182]. The suggested conditions would preclude formulation where the concentration is above a legally applicable limit, unless a derogation is offered.

In the United States, active and intelligent materials fall under the FDA’s food-contact substance framework, which necessitates premarket clearance if the active agent is deemed a “food additive” [178]. Manufacturers should consider possible migrants, decomposition by-products or impurities resulting from chemical interactions between the active agent, the polymer matrix and food components during storage and shelf life [180,183]. Regulatory pathways for active packaging based on phytochemicals can be complicated, since many plant extracts are complex mixtures, rather than single, well-characterized compounds [184,185]. The generally recognized as safe (GRAS) status that could be associated with some essential oils and polyphenols as food additives does not necessarily imply the application as packaging, which should be independently evaluated, especially when they are embedded into novel matrices or nanostructured carriers [186]. Toxicological assessment of phytochemical-functionalized packaging ought to concentrate on the final formulation and the substances migrating into food, rather than the natural origin or biocompatibility of individual ingredients. Migration testing should be tailored to the intended food-contact situation (food composition, time and temperature of food contact, and surface-area-to-food ratio) and intentional phytochemical release, rather than unintended migration. Estimates of dietary exposure are based on food-consumption data and body weight together with migrant concentration in food and appropriate high-consumption groups plus background exposure [187]. Additionally, botanical extracts in food-contact materials should be characterized, including batch-to-batch variability, considering the botanical source, extraction/processing, and relevant contaminants/residual solvents [182]. Chemical characterization and toxicological evaluation of relevant migrants [180,187] should also consider degradation products from formulation, processing, and storage, residual cross-linkers, unreacted materials, and any other non-intentionally added substances. For nanocomposite systems, the release of nanoparticles should also be considered, which separates particulate migration from dissolved ions or molecules and takes account of particle properties and dissolution profile as well as limits of analytical detection; no automatic extrapolation of safety conclusions for bulk materials to nanoscale forms should be made [188]. Thus, compliance with overall migration or a negative cytotoxicity test result should not be considered as evidence of food-contact safety [187,188]. Selected European Union and United States regulatory frameworks relevant to phytochemical-functionalized active packaging are summarized in Table 2, with official sources and distinctions between material-specific migration requirements and substance-specific conditions of use.

### 9.2. Sensory Impact and Consumer Acceptance

One of the most important practical obstacles to the commercialization of phytochemical-loaded films is the possibility of altering the sensory attributes of packaged foods such as odor, taste, color and texture [194,195]. Essential oils are well known to be antimicrobials, but they are also very volatile and aromatic in nature. In packaging, they can cause a strong herbal, spicy or medicinal note, which may be undesirable for some products [162,194]. For example, cinnamon and oregano oils have strong bioactivity, but at high loadings (>1–2%) can mask the natural flavor of sensitive foods such as dairy, fresh produce, or mild meats [163].

Moreover, the polyphenol-rich extracts, especially with intense coloration (anthocyanins, curcumin, and chlorophyll) may cause a strong staining/discoloration of the film or the food surface, which may be considered as negative by consumers, even if the product is safe and nutritious [195,196]. Sensory impacts can be mitigated by encapsulation (e.g., cyclodextrins, liposomes, and nanoparticles) to reduce volatility and mask odor, controlled release formulations to reduce burst migration, and optimization of loading levels to balance efficacy and sensory thresholds [196]. Studies on consumer acceptance are limited but suggest that the adoption of active packaging is influenced by perceived benefits such as extended shelf life, reduced food waste and natural ingredients, which must outweigh concerns regarding chemical migration, allergenicity or flavor change. Clear labelling, education on safety and functionality, and alignment with clean-label trends (plant-derived, non-GMO, and organic) can bolster consumer trust and market penetration [197].

### 9.3. Scalability and Cost-Effectiveness

Solvent casting is commonly used in laboratory research but it is energy intensive, slow and difficult to scale-up for industrial production which limits its commercial viability [198,199]. Thermoforming and extrusion provide scalable options for thermoplastic biopolymers (PLA, PHBV, and PBAT), but high processing temperatures (150–200 °C) potentially impact heat-sensitive phytochemicals, necessitating protective carriers or the choice of thermostable compounds [113,200]. Cost remains a potential barrier to commercialization, but comparisons with conventional plastics should distinguish raw-material prices from finished-film production costs and specify the polymer grade, production scale, location, and assessment period. A single cost multiplier should therefore not be generalized across starch-, cellulose-, and PLA-based packaging systems [201]. Moreover, the requirement for specialized equipment, energy-intensive drying processes, and limited supply chains for agricultural feedstocks further restrict scalability and economic competitiveness. Such barriers are particularly severe for small and medium-sized enterprises (SMEs) and need policy support, collaborative R&D and investment in shared manufacturing infrastructure [201]. Recent advances such as continuous extrusion, multilayer coextrusion and roll-to-roll coating can decrease costs and increase throughput, but these technologies need further optimization in phytochemical-loaded systems to ensure uniform dispersion and controlled release [202,203].

### 9.4. Environmental Life-Cycle Considerations

Biodegradable and bio-based packaging materials are often marketed as more environmentally friendly than conventional plastics, but their actual environmental impacts depend on a number of factors, including the source of their feedstocks, the energy used during production, the waste management systems in place at end-of-life, and the conditions of biodegradation [204]. Reported greenhouse-gas benefits should be interpreted within the specific life-cycle assessment, including its functional unit, system boundaries, polymer and reference material, feedstock source, energy mix, allocation assumptions, and end-of-life scenario; they should not be generalized to all bio-based packaging systems [205,206].

Moreover, bio-based plastics can lead to greater damage to ecosystems through land-use change, water use and loss of biodiversity when the feedstocks are derived from intensive agriculture without sustainability measures [207,208]. Comparisons between composting and incineration should identify the impact category assessed and the assumptions concerning waste treatment and energy recovery, rather than express environmental performance as a single universal percentage. However, infrastructure for industrial composting is not readily accessible in many parts of the world, and the rate of degradation may not be complete in a reasonable timeframe with home composting [209,210]. Future studies should address feedstock diversification (agricultural by-products, algae, and microbial fermentation), energy-efficient processing (renewable electricity and process intensification), design for circularity (reuse, recycling, and controlled biodegradation) and consumer education on proper disposal to maximize environmental benefits.

## 10. Future Perspectives

The intersection of phytochemistry, polymer science and food technology has resulted in the creation of phytochemical-functionalized smart polymer films as a promising solution to the dual challenges of food preservation and environmental sustainability. The present review has shown that substantial progress has been made in the understanding of the molecular mechanisms of phytochemical bioactivity, in the design of tailor-made polymeric matrices, in the construction of controlled release systems and in the validation of functional performance in real food systems. However, there are several critical knowledge gaps and technological barriers that need to be overcome to realize the full capabilities of these materials at commercial scale.

### 10.1. Knowledge Gaps and Research Priorities

First, there is an urgent need for standardized testing protocols that relate in vitro assays to real-food performance. DPPH, ABTS and microbial inhibition assays provide useful screening data, but they often do not predict behaviour in complex food matrices, where pH, water activity, fat level, and competing reactions affect bioactive stability and efficacy [211,212]. Future studies should aim to utilize standardized food model systems and accelerated shelf-life testing protocols that allow for cross-study comparisons and meta-analyses to enable evidence-based formulation design [212]. Second, the quantitative structure–function relationships remain underexplored, especially for complex botanical extracts with tens to hundreds of phytochemicals. Structure–activity relationships of individual compounds (gallic acid, quercetin, carvacrol, and eugenol) are well characterized, but the synergistic and antagonistic interactions of extracts and between extracts and polymer matrices are poorly understood [213]. Advanced analytical techniques, such as metabolomics, molecular docking, and in situ spectroscopy, in combination with machine learning approaches can help to elucidate these interactions and to develop predictive models for bioactive release, stability and performance [214].

Third, multi-stimuli-responsive systems that integrate multiple triggers (pH, temperature, humidity, and spoilage volatiles) within a single platform constitute an emerging frontier with transformative potential [215]. Stimuli-responsive designs are usually designed to respond to only one trigger, limiting their adaptability to dynamic spoilage environments. Inspired by biomedical drug delivery technologies, dual- or triple-responsive hydrogels, nanogels, and microcapsules could enable adaptive, feedback-controlled release, mimicking food deterioration kinetics [103,106]. Fourth, smart packaging that combines active functioning with sensing and communicating abilities allows for real-time quality monitoring and consumer involvement [216]. RFID tags or QR codes linked to smartphone apps can offer recipes, storage tips and traceability, thus improving customer confidence and reducing food waste.

### 10.2. Technological and Commercialization Considerations

To this end, innovative scalable manufacturing techniques, from lab-scale solvent casting to industrial extrusion, coextrusion and roll-to-roll coating [217], need to be developed. Continuous processing offers advantages in terms of throughput, consistency and cost, but requires optimization for phytochemical stability and controlled-release performance [218]. Hybrid methods combining extrusion for the base film manufacture with post-extrusion coating or impregnation for the incorporation of bioactives can be feasible strategies for bridging the gap between scalability and functionality. Cost-reduction strategies should be evaluated for the specific formulation and manufacturing process rather than assuming a uniform price premium for all biopolymer feedstocks. The valorization of agricultural and food processing by-products (e.g., fruit peels, seed meals, and spent grains) as sources of biopolymers and phytochemicals is in line with circular economy principles and reduces raw material costs. Joint research and development, public–private partnerships, and policy-driven incentives such as subsidies, tax credits, and green procurement can accelerate the pace of technology transfer and market entry, particularly for financially constrained small and medium-sized enterprises [218].

Harmonization of regulations among jurisdictions would facilitate international trade and reduce the compliance burden for manufacturers seeking to obtain approval in more than one market. The EU and FDA frameworks are built on the same fundamental safety principles, meaning that differences in migration testing protocols, substance lists and GRAS acknowledgment lead to complexity for botanical extracts and novel nanomaterials. Mutual recognition agreements and international task forces can help to speed up approval processes and reduce time-to-market.

### 10.3. Environmental and Societal Implications

Environmental life-cycle assessments need to move beyond the simple “bio-based equals sustainable” narrative and consider where the feedstock comes from, how much energy is used in the production process, what infrastructure is required to handle the end of life and the effects on ecosystems. Prioritizing non-food feedstocks (agricultural residues, algae, and microbial fermentation), integrating renewable energy and designing for circularity (reuse, recycling, and controlled biodegradation) will be essential to maximize net environmental benefits [219,220]. Active packaging can contribute significantly to food security and waste reduction from a societal perspective, especially in regions with limited cold chain infrastructure and high post-harvest losses [221]. Phytochemical-functionalized films can extend shelf life and preserve nutritional quality, thus reducing the need for synthetic preservatives, refrigeration, and over-packaging, in line with consumer demand for clean-label, sustainable products.

## 11. Conclusions

Phytochemical-functionalized smart polymer films are a paradigm shift from passive containment toward active and intelligent food preservation. The materials offer multifunctional solutions by combining the antioxidant and antimicrobial power of plant-derived bioactives with the tunable properties of biopolymer matrices and advanced delivery systems, in a simultaneous response to the challenges of food safety, quality, shelf life and environmental sustainability.

This unique review presents a systems-level framework connecting phytochemical structure, polymer architecture, release kinetics and real-food performance to actionable design guidelines for researchers and developers. While challenges remain in standardization, scalability, cost, regulation and environmental optimization, there is no question that the rapid pace of innovation driven by the interdisciplinary collaboration and increasing market demand provides a promising path toward commercialization.

With the further development of the field, future work should focus on standardized testing and prediction modelling, integration of multi-stimuli-responsive and intelligent packaging, scalable manufacturing and cost reduction, regulatory harmonization, and holistic life-cycle optimization. Addressing these priorities will enable the scientific and industrial communities to harness the full capabilities of phytochemical-functionalized smart films, contributing to a more sustainable, resilient and health-conscious global food system.

## Figures and Tables

**Table 1:** Representative case studies of phytochemical-functionalized films in real food systems.

Sl. No.	Food Product	Polymer Matrix/System	Phytochemical or Active Component	Packaging Format	Reported Application Outcome and Interpretation	Ref
1	Chilled pork	Chitosan-based system	Blackberry anthocyanins and tea polyphenols	Edible coating applied directly to pork	Combined coating suppressed microbial growth and lipid oxidation and supported quality retention during refrigerated storage. This is a direct food-preservation application, not a standalone-film study.	[161]
2	Pork	L100-polymer-based coaxially electrospun system	Cinnamon essential oil	Preformed core–shell fibrous film	Demonstrated pH-dependent essential-oil release and antibacterial functionality for meat preservation. The release response was investigated directly rather than inferred from a color change.	[19]
3	Pork	Chitosan film containing shellac-based nanocapsules	Cinnamaldehyde	Preformed composite film	Nanocapsule-mediated release responded to the alkaline microenvironment associated with meat deterioration. The composite film exhibited antimicrobial functionality and improved pork preservation.	[135]
4	Fresh minced lamb	Poly(lactic acid)/nanochitosan composite	Polylophium involucratum essential oil	Preformed composite film	Improved microbial, oxidative, and sensory quality retention. Preservation performance depended on the essential-oil concentration in the composite formulation.	[162]
5	Fresh beef	Sodium alginate nanocomposite	Cumin essential oil and titanium dioxide nanoparticles	Standalone cast film used to wrap beef	Reduced microbial growth and lipid oxidation and improved sensory quality during refrigerated storage. Preservation was evaluated against unwrapped beef and films containing individual formulation components.	[163]
6	Domiati cheese	Carboxymethyl chitosan nanoparticle-based system	Pomegranate peel extract	Edible nanocoating applied directly to cheese	Improved microbial quality during refrigerated storage. Antioxidant activity measured in coating-material assays should be distinguished from oxidation outcomes measured directly in cheese.	[164]
7	Cheese-packaging application	Chitosan–procyanidin composite	Procyanidins	Standalone solution-cast film	Demonstrated antioxidant, antimicrobial, and pH-responsive properties relevant to cheese packaging. These material-level functions should not be interpreted as evidence of stimulus-triggered antimicrobial release or a quantified cheese shelf-life extension.	[29]
8	White button mushrooms	Poly(lactic acid) containing mesoporous silica nanoparticles	Clove essential oil loaded into mesoporous silica	Standalone film fabricated into packaging bags	Sustained essential-oil release supported retention of mushroom quality, including reduced microbial deterioration and enzymatic browning during refrigerated storage.	[95]
9	Pacific white shrimp	Poly(butylene adipate-co-terephthalate)/poly(lactic acid) blend	Carvacrol, citral, or α-terpineol	Standalone blown-extruded film	Active formulations inhibited microbial growth and lipid oxidation in shrimp. The three active compounds were investigated as formulation alternatives and should not be presented as a single combined treatment.	[88]
10	Raspberries	Nanofibrillated cellulose	Thyme, cinnamon, or oregano essential oils; selected combinations also investigated	Preformed film insert attached inside the package lid for non-contact delivery	Essential-oil-loaded inserts suppressed fungal deterioration during refrigerated storage. Performance depended on oil identity, dose, and treatment combination. The system was an active insert, not a directly applied fruit coating.	[32]
11	Strawberries	Polyvinylpyrrolidone/shellac core–sheath system	Eugenol	Preformed coaxially electrospun fibrous film	Investigated controlled eugenol delivery for maintaining post-harvest strawberry quality and extending storage life. The fibrous film should be distinguished from an edible coating applied directly to the fruit.	[103]
12	Mango fruits	Sodium alginate incorporating a cellulose-nanocrystal-stabilized Pickering emulsion	Ginger essential oil	Composite film; food-application configuration requires confirmation from the original methods	Pickering emulsion system improved the sustained-release properties on ginger essential oil of sodium alginate film and delayed the post-harvest senescence of mango fruits.	[99]
13	Blackberries	Poly(lactic acid) nanofibers covered with poly(N-isopropylacrylamide)	Lemon essential oil	Preformed thermoresponsive nanofiber membrane	Temperature-responsive regulation of essential-oil release supported blackberry preservation under changing temperatures. The responsive release function was associated with the thermosensitive polymer layer.	[128]
14	Fresh fruits Strawberries	Humidity-responsive, gas-generating nanofiber system	Thyme essential oil	Preformed active nanofiber film	Humidity-triggered thyme essential-oil release inhibited microbial growth, reduced weight loss, and maintained strawberry firmness, supporting shelf-life extension.	[125]

**Table 2:** Regulatory frameworks and migration limits for active packaging materials.

Sl. No.	Jurisdiction	Principal Regulatory Framework	Key Requirements	Migration and Exposure Assessment	Official Sources	Ref
1	European Union	Regulation (EC) No. 1935/2004 provides the general food-contact framework; Regulation (EC) No. 450/2009 addresses active and intelligent materials; Regulation (EU) No. 10/2011 applies to plastic materials and articles within its scope.	Finished materials must satisfy applicable safety requirements under normal or foreseeable use. Intentionally released active substances must comply with the relevant provisions applicable to food. Active packaging must not mask spoilage in a way that misleads consumers.	For plastics within the scope of Regulation (EU) No. 10/2011, Article 12 specifies an OML of 10 mg/dm^2^, with a specific 60 mg/kg food-simulant provision for articles intended for foods for infants and young children. Applicable substance-specific migration limits and restrictions must also be met. Released active substances are treated separately under Article 9 of Regulation (EC) No. 450/2009.	European Parliament and Council (2004), Articles 3–4; European Commission (2009), Article 9; European Commission (2011, as amended), Articles 11–12 and relevant annexes. 1109	[189,190,191]
2	United States	Federal Food, Drug, and Cosmetic Act and applicable FDA regulations and authorizations, including food-contact notifications, food-additive regulations, and Threshold of Regulation exemptions, as appropriate.	The applicable legal basis must cover the substance’s identity, intended use, specifications, and conditions of use. Effective food-contact notifications are specific to the identified manufacturer or supplier and the authorized use.	Assessment considers migrating components, cumulative dietary exposure, and toxicological evidence supporting safe exposure. Apply the restrictions in the relevant regulation, notification, or exemption rather than assuming an EU-style universal OML.	U.S. Food and Drug Administration (2025), “Understanding How the FDA Regulates Substances that Come into Contact with Food”; 21 CFR Part 170, Subpart D. 1092	[192,193]

Abbreviations: CFR = Code of Federal Regulations; EU = European Union; FDA = U.S. Food and Drug Administration.

## Data Availability

No new data were created or analyzed in this study. Data sharing is not applicable to this article.

## Abbreviations

The following abbreviations are used in this manuscript:

ABTS	2,2′-azino-bis(3-ethylbenzothiazoline-6-sulfonic acid)
Ag	Silver
AO	Antioxidant
APFs	Antioxidant packaging films
BA	Black anthocyanins
BOEO	Bitter orange essential oil
BPA	Bisphenol A
CD	Cyclodextrin
CEO	Cinnamon essential oil
CMC	Carboxymethyl cellulose
CNC	Cellulose nanocrystal/nanocellulose
CNF	Cellulose nanofiber
CS	Chitosan
CS/BA/TP	Chitosan/black anthocyanins/tea polyphenols coating
CuO	Copper(II) oxide
DE-PEG	Diepoxy-polyethylene glycol
DPPH	2,2-diphenyl-1-picrylhydrazyl
EB	Elongation at break
EC	European Commission
EFSA	European Food Safety Authority
EO	Essential oil
FCM	Food-contact material
FRAP	Ferric reducing antioxidant power
GHG	Greenhouse gas
GNP	Graphene nanoplatelet
GRAS	Generally recognized as safe
HAT	Hydrogen atom transfer
LCST	Lower critical solution temperature
LCA	Life-cycle assessment
LOO-	Lipid peroxyl radical
LOOH	Lipid hydroperoxide
MC	Methylcellulose
MCC	Microcrystalline cellulose
MDA	Malondialdehyde
MIC	Minimum inhibitory concentration
MMT	Montmorillonite (clay)
NPs	Nanoparticles
OML	Overall migration limit
OP	Oxygen permeability
ORAC	Oxygen radical absorbance capacity
PBAT	Poly(butylene adipate-co-terephthalate)
PE	Polyethylene
PEO	Polyethylene oxide
PHB	Polyhydroxybutyrate
PHBV	Poly(3-hydroxybutyrate-co-3-hydroxyvalerate)
PLA	Polylactic acid
PNIPAM	Poly(N-isopropylacrylamide)
PPE	Pomegranate peel extract
RH	Relative humidity
ROS	Reactive oxygen species
SET	Single-electron transfer
SML	Specific migration limit
SPI	Soy protein isolate
TA	Titratable acidity
TBARS	Thiobarbituric acid reactive substances
TMC	Transition metal chelation
TSS	Total soluble solids
TVB-N	Total volatile basic nitrogen
TVC	Total viable count
UV	Ultraviolet
WVP	Water-vapor permeability
ZnO	Zinc oxide
